# Capturing clinically relevant Campylobacter attributes through direct whole genome sequencing of stool

**DOI:** 10.1099/mgen.0.001284

**Published:** 2024-08-30

**Authors:** Bilal Djeghout, Thanh Le-Viet, Leonardo de Oliveira Martins, George M. Savva, Rhiannon Evans, David Baker, Andrew Page, Ngozi Elumogo, John Wain, Nicol Janecko

**Affiliations:** 1 Quadram Institute Bioscience, Norwich Research Park, Norwich NR4 7UQ, UK; 2 Eastern Pathology Alliance, Norfolk and Norwich University Hospital, Norwich NR4 7UY, UK; 3 Norwich Medical School, University of East Anglia, Norwich NR4 7TJ, UK

**Keywords:** *Campylobacter*, clinical samples, metagenome-derived genomes

## Abstract

*Campylobacter* is the leading bacterial cause of infectious intestinal disease, but the pathogen typically accounts for a very small proportion of the overall stool microbiome in each patient. Diagnosis is even more difficult due to the fastidious nature of *Campylobacter* in the laboratory setting. This has, in part, driven a change in recent years, from culture-based to rapid PCR-based diagnostic assays which have improved diagnostic detection, whilst creating a knowledge gap in our clinical and epidemiological understanding of *Campylobacter* genotypes – no isolates to sequence. In this study, direct metagenomic sequencing approaches were used to assess the possibility of replacing genome sequences with metagenome sequences; metagenomic sequencing outputs were used to describe clinically relevant attributes of *Campylobacter* genotypes. A total of 37 diarrhoeal stool samples with *Campylobacter* and five samples with an unknown pathogen result were collected and processed with and without filtration, DNA was extracted, and metagenomes were sequenced by short-read sequencing. Culture-based methods were used to validate *Campylobacter* metagenome-derived genome (MDG) results. Sequence output metrics were assessed for *Campylobacter* genome quality and accuracy of characterization. Of the 42 samples passing quality checks for analysis, identification of *Campylobacter* to the genus and species level was dependent on *Campylobacter* genome read count, coverage and genome completeness. A total of 65% (24/37) of samples were reliably identified to the genus level through *Campylobacter* MDG, 73% (27/37) by culture and 97% (36/37) by qPCR. The *Campylobacter* genomes with a genome completeness of over 60% (*n*=21) were all accurately identified at the species level (100%). Of those, 72% (15/21) were identified to sequence types (STs), and 95% (20/21) accurately identified antimicrobial resistance (AMR) gene determinants. Filtration of stool samples enhanced *Campylobacter* MDG recovery and genome quality metrics compared to the corresponding unfiltered samples, which improved the identification of STs and AMR profiles. The phylogenetic analysis in this study demonstrated the clustering of the metagenome-derived with culture-derived genomes and revealed the reliability of genomes from direct stool sequencing. Furthermore, *Campylobacter* genome spiking percentages ranging from 0 to 2% total metagenome abundance in the ONT MinION sequencer, configured to adaptive sequencing, exhibited better assembly quality and accurate identification of STs, particularly in the analysis of metagenomes containing 2 and 1% of *Campylobacter jejuni* genomes. Direct sequencing of *Campylobacter* from stool samples provides clinically relevant and epidemiologically important genomic information without the reliance on cultured genomes.

**Impact Statement**

Direct sequencing of the intestinal pathogen *Campylobacter* from clinical stool samples is difficult due to the low abundance of *Campylobacter* cells and high levels of DNA from other microbes and host cells; this impairs the quality of *Campylobacter-*specific sequence outputs. We identified sequencing metrics to evaluate the reliability of direct sequencing (metagenomics) for characterizing key attributes of *Campylobacter* that are important to clinicians and epidemiologists. Characterization to species level, sequence type and antimicrobial resistance genotypes were obtained from metagenome-derived genomes when the *Campylobacter* genome assembly reached >60% completeness and contained more than 12 500 *Campylobacter* reads with a sequencing coverage of >5. The resulting *Campylobacter* attributes were accurately validated against culture-derived genomes of corresponding stool samples. Sequencing quality was improved when stool samples were physically filtered before DNA extraction. *Campylobacter* genome assemblies produced nearly closed genomes of high quality and accurate attribute identification when used with long-read sequencing and targeted genome (adaptive) sequencing on stool metagenomes spiked with 1–2% *Campylobacter*. These findings highlight the feasibility of *Campylobacter* characterization through direct metagenomic sequencing of stool samples and methods that could be optimized further, for stool-to-genome sequencing in clinical and epidemiological investigations.

## Data Summary

All relevant supporting data are available in the accompanying supplementary data files. The online version of this article contains five supplementary tables and three supplementary figures. All *Campylobacter* isolate genomes and metagenome sequences are available in the National Center for Biotechnology Information (NCBI) Sequence Read Archive (SRA) under the Bioproject accession numbers PRJNA1046283, PRJNA797426 and PRJNA1049393. SRA accession numbers and associated metadata for isolate genomes and metagenomes are included in Table S1, available in the online version of this article. A comprehensive compilation of codes and parameters for all computational tools and analysis steps is deposited on GitHub (https://github.com/quadram-institute-bioscience/2024-campymags).

## Availability of Data and Materials

All supporting data, code and protocols are included in the article or are available as supplementary data files. The online version of this article contains three supplementary figures (Figs S1–S3) and five supplementary tables (Tables S1–S5). All sequenced *Campylobacter jejuni* isolate data are available in the National Center for Biotechnology Information (NCBI) Sequence Read Archive under the Bioproject accession numbers PRJNA1046283, PRJNA797426 and PRJNA1049393. Sequence read archive (SRA) accession numbers and associated metadata can be found in the supplementary material of this study (Table S1). All data analysis codes, scripts and pipelines are deposited on GitHub (https://github.com/quadram-institute-bioscience/2024-campymags/tree/main).

## Introduction

*Campylobacter* is the leading cause of bacterial gastroenteritis worldwide with an estimated 400–500 million cases reported globally each year [1,2]. The predominant species responsible for human illness are *Campylobacter jejuni* and *C. coli*. An infection in humans requires a low infectious dose that is estimated to be between 500 and 800 organisms and both species account for a small proportion of the overall gut microbiome population during peak infection [3,5]. Despite the low abundance within the gut, the infection manifests into clinical symptoms ranging from mild gastroenteritis to severe life-threatening sequelae [3]. Diagnosis of campylobacteriosis requires a clinical evaluation and laboratory testing of stool that most commonly includes a multi-pathogen rapid PCR assay without culturing for presumptive isolates [6,7]. If testing by culture is conducted, the characterization of *Campylobacter* can be challenging due to the fastidious nature of the pathogen in laboratory growth conditions and the low abundance of the organism in diarrhoeal stool [8,9]. Culture-independent testing for diagnostic identification provides a fast and accurate detection result to the genus or species level, but despite the advantages of PCR-based methods, the assays lack comprehensive genotypic information on *Campylobacter* characteristics leading to the loss of clinically relevant attributes that may impact effective infection management [10,11]. This in turn impacts the knowledge base for clinical decisions, public health management and intervention strategies for circulating and emerging *Campylobacter* strains in the regional human population [12].

One way to mitigate the genomic knowledge gap is to use culture-independent direct whole genome sequencing of stool (metagenomic extraction) to provide *in silico Campylobacter* genotype attributes important for clinical and epidemiological purposes such as species, sequence types (STs) and antimicrobial resistance (AMR) determinants. Metagenomic analysis of gut microflora can provide the population structure of the microbial population in the gut, but metagenome extraction and subsequent analysis can have limitations of precision and coverage depth [13,16]. Early metagenomic applications were utilized in parallel with traditional outbreak investigations and were successful in identifying *Escherichia coli* O104:H4, yet the sensitivity of analysis, speed, bioinformatic workflow complexity and cost limited its wider implementation [17]. Another study compared the gene variabilities of pathogenic *E. coli* isolates from eight diarrheal samples against their corresponding metagenome-derived genomes (MDGs) recovered from the same metagenomic data set [18]. The findings revealed that even MDGs with high completeness estimates (near 95%) captured 77% of the population core genes and 50% of the population variable genes on average. Additionally, around 5% of the genes in these MDGs were identified as missing in the isolate, suggesting errors in the genome-binning step [18]. The assessment of clinically important attributes was not investigated. In a metagenome profiling study of 100 infant diarrhoeal stool samples, uncultured *Campylobacter* species were detected in 36 samples [19]. Informatic tools and databases have improved the sensitivity and specificity of pathogen identification with the cost–benefit analysis approaching affordability when culture, PCR, sequencing and subtyping assays are included [20]. A high sequencing depth is still needed for sufficient coverage of low-abundance organisms to recover MDGs from the metagenomes of stool [21], yet it is unclear what coverage is required for *Campylobacter* genomes. As an example, the study successfully obtained nearly 8000 draft-quality genomes from more than 1500 metagenomes. Notably, all genomes were estimated to be at least 50% complete, with almost half being over 90% complete and having less than 5% contamination. This achievement is significant considering challenges such as community variation, uneven genome coverage and fragments from uncultivated species, which can hinder genome assembly and limit interpretation in pathogen-focused metagenomics [22]. The scale and quality of the dataset demonstrated the efficiency of recovering genomes from metagenomes in exploring the microbial dark matter and expanding our understanding of the tree of life [22]. Furthermore, accurate functional inference from MDGs requires sufficient genome completeness, or conclusions may lead to an underestimation of the functional capacity of the genomes [23]. One way to compensate would be to use more relaxed core gene thresholds, select gene prediction tools considering fragmented genes, and use mixed datasets that include both MDGs and complete genomes to alleviate the accuracy loss due to the use of MDGs [24].

Short-read metagenomic sequencing, which typically uses paired-end reads of 150 bases, facilitates comprehensive coverage of microbial communities [25]. However, technological advances now support read lengths of up to 300 bases on platforms such as the MiSeq, potentially further improving assembly quality by enhancing the resolution of complex genomic regions and larger structural variations [26]. Long-read sequencing has the potential to enable the assembly of complete genomes for complex feature detection, but the cost remains prohibitory with a higher DNA volume requirement and a higher base-calling error [27].

Oxford Nanopore Technologies (ONT) sequencing has emerged as a powerful alternative due to its ability to generate long reads and its portable nature [28]. A pivotal advancement in ONT sequencing is the implementation of adaptive sequencing, a technique that dynamically selects DNA fragments for sequencing in real time based on predefined criteria [29]. This innovative method has been shown to be a valuable tool for enriching low-abundance species in metagenomic samples [30]. Adaptive sampling has been effectively used in workflows for diagnosing bacterial pathogens and antimicrobial resistance genes (ARGs) [30]. By selectively depleting human host DNA and enriching microbial DNA in the processing stages of sample preparation, this method can significantly increase the microbial sequence yield [30]. By focusing sequencing efforts on specific genomic regions of interest, adaptive sampling enhances the specificity of pathogen identification in clinical samples [30,31].

Sequence quality metrics are pivotal in guaranteeing the accuracy and reliability of the metagenomic data. N50 and genome completeness are significant metrics for assessing the quality of metagenomic assemblies [32]. Higher values of these metrics indicate a more complete assembly, which is more likely to represent the original genomes in the metagenomic data [32]. However, these metrics alone do not capture the correctness of the assemblies. To provide a more comprehensive evaluation, BUSCO (Benchmarking Universal Single-Copy Orthologues) scores, which assess the presence and completeness of conserved orthologues in the assemblies, is an important tool to consider [33]. This is particularly important for nanopore-only assemblies, which can retain frameshifts and other errors due to homopolymeric repeats, even after polishing and proofreading [34,35]. Including BUSCO scores ensures that the assemblies are not only contiguous but also accurate and reliable [36].

In this context, this study aimed to evaluate the sequence quality metrics of *Campylobacter* for clinical and public health purposes, through metagenome analysis, and validate the accuracy of *Campylobacter* MDGs against genomes cultured from the same stool samples.

## Methods

### Stool sample collection

The study was conducted under the ethics approval of the University of East Anglia Research Ethics Committee (Ref. 2018/19-159). Human tissue (stool) research was conducted under Norwich Biorepository licence NRES number – 19/EE/0089; IRAS Project ID – 259062 approved by the UK’s Human Tissue Authority (HTA). The National Health Service (NHS) Eastern Pathology Alliance (EPA) network diagnostic laboratory in Norwich, UK, was the sole source diagnostic laboratory participating in this study.

**Fig. 1.**
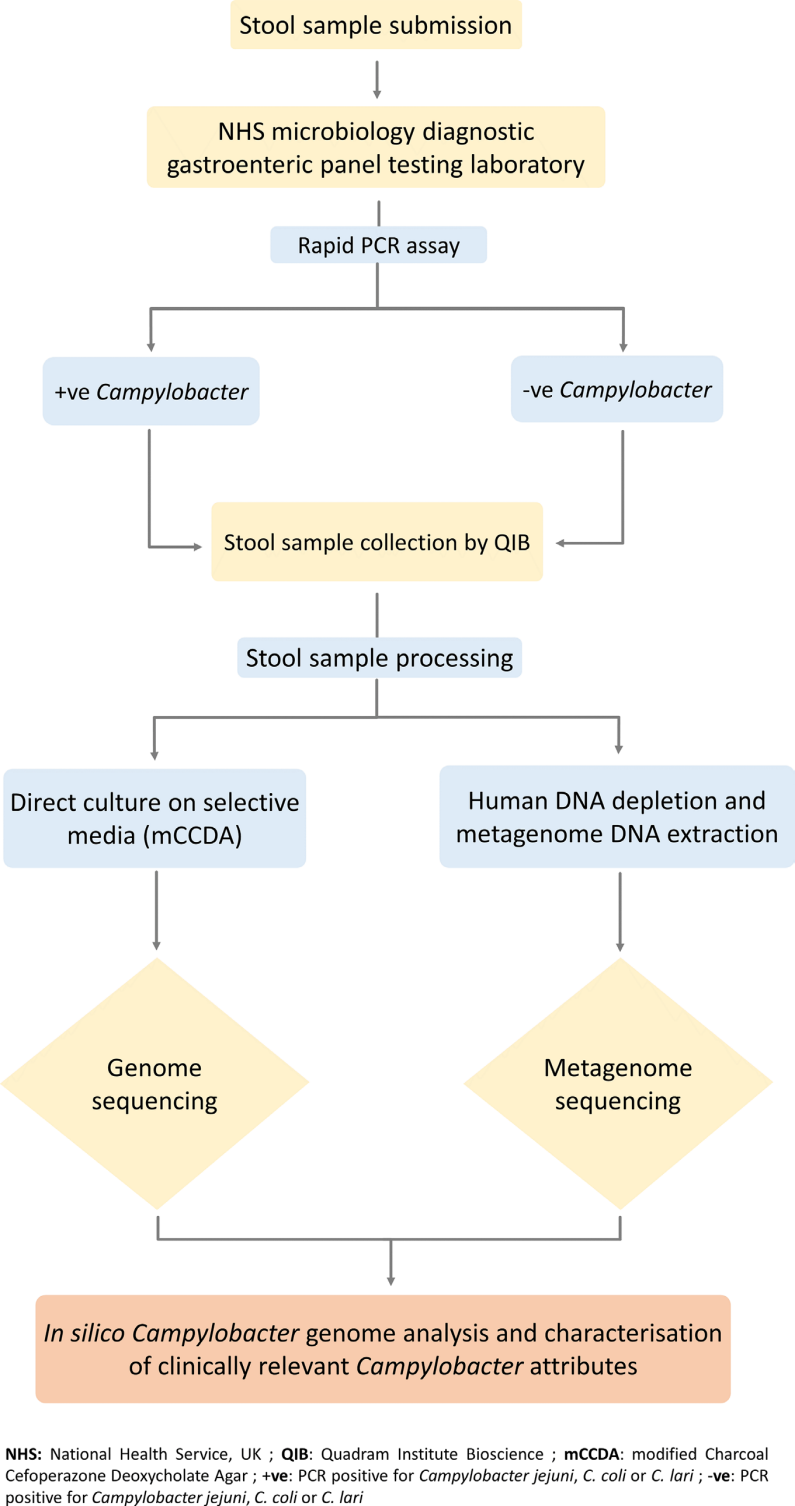
Overview workflow of stool samples for the analysis of *Campylobacter* spp. from patients with gastroenteritis.

As part of the routine infectious intestinal disease screening, the EPA laboratory tested submitted stool samples for *Campylobacter* spp. and other infectious intestinal pathogens using a rapid automated PCR-based culture-independent testing panel (Gastro Panel 2, EntericBio Serosep) between August 2020 and June 2022. For this study, the inclusion criteria comprised PCR-positive (PCR+) results for *Campylobacter* in stool samples collected within 5 days of submission, with a sample volume of >5 ml, and a stool Bristol scale value ranging from 3 to 7. Negative controls were defined as samples PCR-negative (PCR-) for *Campylobacter* while meeting all other study inclusion parameters. After routine testing was complete, the surplus of stool samples meeting study inclusion criteria were collected monthly by the research team to represent *Campylobacter* infections throughout the year. Up to 5 ml aliquots of stool sample were transported to the Quadram Institute Bioscience testing laboratory in Norwich using standard transport protocols for human tissue. Samples were processed for stool metagenome DNA extraction and *Campylobacter* isolation using adapted processing and isolation methods (Fig. 1).

### Stool sample preparation

#### Unfiltered stool

Stool samples prepared without filtration are referred to as unfiltered samples. To minimize the amount of human DNA accounting for sequencing capacity, a method of host DNA depletion was adapted to analyse stool samples [37]. In brief, human host DNA was digested by adding 200 µl of buffer (5.5 M NaCl and 100 mM MgCl_2_ in nuclease-free water) to 200 µl of stool. Next, 35 µl of 1% saponin (Tokyo Chemical Industry UK) was added to the stool buffer mixture, followed by the addition of 10 µl of HL-SAN DNase (Articzymes). The samples were mixed thoroughly and incubated at 37 °C with shaking at 800 r.p.m. for 20 min. The host-DNA depleted samples were washed with 300 µl of PBS [NaCl (58.44 g mol^–1^), KCl (74.55 g mol^–1^), Na_2_HPO_4_ (141.96 g mol^–1^ and KH_2_PO_4_ (136.09 g mol^–1^. Following the wash step, samples were centrifuged at 13 000 r.p.m. for 5 min, and the supernatant was carefully discarded.

#### Filtered stool

Filtration of stool was conducted before DNA extraction for samples containing a sufficient amount of stool. This subset of samples was representative of the whole study sample set in terms of Bristol stool scale and collection date. An aliquot of 1 g or 1 ml of stool was homogenized in 9 ml of peptone buffer solution (Oxoid). The sample was filtered using 0.65 µm pore size syringe filters (Sartorius AG). Up to 8 ml of sample filtrate was concentrated by centrifugation at 13 000 r.p.m. for 5 min. The supernatant was discarded, and the remaining pellet was considered the input sample for host DNA depletion as described previously in raw stool preparation and was considered for metagenome DNA extraction.

#### Metagenomic DNA extraction

The host-depleted stool pellet from both methods (unfiltered and filtered) was considered as input stool and underwent metagenomic DNA extraction. The Shoreline Breaker DNA extraction system (Shoreline Biome) was employed for 19 samples, and the Maxwell RSC Fecal Microbiome DNA Kit (Promega) was used for 23 samples. Metagenome DNA extraction was performed following the respective manufacturer’s instructions for each kit. DNA quantification was carried out using a Qubit fluorometer with dsDNA quantification high-sensitivity kit (Life Technologies).

#### *Campylobacter* quantification in stool

A quantitative PCR (qPCR) assay was conducted on all metagenome DNA extracts, using the LightCycler 480 Probes Master kit on the LightCycler 480 Instrument II (software LCS480 v1.5.0.39) (Roche Diagnostics). The target gene for *Campylobacter* quantification was the *cadF* gene previously described [38,39]. In brief, each reaction contained a 20 µl reaction mixture comprising 2 µl of DNA sample, 10 µl of a primer–probe master mix, 7 µl of nuclease-free water, 0.4 µl of *cadF* forward and reverse primers (10 µM), and 0.2 µl of *cadF* probe (10 µM). The qPCR cycling conditions consisted of a pre-amplification step at 95 °C for 10 min to initiate the reaction. The amplification phase comprised 45 cycles, with each denaturation cycle at 95 °C for 15 s, followed by annealing and extension at 55 °C for 1 min. Subsequently, a cooling step was performed at 44 °C for 30 s to facilitate post-reaction stabilization. Samples with CT values (cycle threshold) falling within the range of 1–40 cycles were considered positive for *Campylobacter*, indicating the presence of the target gene (*cadF*) in the sample. Conversely, samples with CT values outside this range, i.e. above 40 cycles, were classified as negative for *Campylobacter*, indicating the absence of detectable *Campylobacter* DNA in the sample.

#### Short-read metagenome DNA library preparation and sequencing

Metagenome DNA libraries were constructed using an Illumina DNA Prep (M) Tagmentation kit following the manufacturer’s instructions (Illumina). Paired-end indexed libraries were sequenced on the Illumina NovaSeq PE150 platform according to the manufacturer’s instructions (Novogene) aiming for at least 10 GB of depth from each sample. Quality control (QC) was conducted on the metagenome DNA libraries before sequencing. QC for each sample included sample quantification using Qubit fluorometry to determine DNA concentration, as well as assessing insert size and molar concentration by using a Tapestation 4200 system with D5000 reagents and tape (Agilent).

#### Short-read metagenome-derived *Campylobacter* data analysis

Raw sequence reads of each sample were stored on an in-house instance of IRIDA [40]. As an initial assessment of *Campylobacter* presence in the metagenomes, we estimated their completeness and quality. Prior to all downstream data analysis, any remaining human DNA reads were removed and eliminated from the dataset using Kraken2 (v2.1.1) [41] and a custom-built database of human genomes with no masking confidence set to 0.1. fastp (v0.22.0) [42] was used to filter reads for quality, remove adapters and trim reads. Taxonomic abundances within each metagenome were estimated using Kraken2 (v2.1.1) [41] and Bracken (v2.6.0) [43] with the database k2_pluspf_20210517 (https://benlangmead.github.io/aws-indexes/k2) to generate a microbiological profile of specimens. *Campylobacter* reads classified up to the genus level were extracted from the Kraken2 classification output, and hence the reads were treated as isolate reads and assembled using shovill (v1.1.0) (https://github.com/tseemann/shovill). Genome completeness of *Campylobacter* was assessed using BUSCO (v3.0.2) [44] and CheckM (v1.0.11) [45]. QUAST (v5.0.2) [46] was used to evaluate genome size distribution. Multi-locus sequence type (MLST) (v2.16.1) [47] calling was conducted using the PubMLST database to assess the ST of *Campylobacter* genomes assembled from the metagenome-derived reads. *In silico* identification of AMR determinants and gene mutations was conducted with the AMRFinderPlus (v3.11.4) [48] database. *Campylobacter* genomes assembled from metagenomes are therefore defined as *Campylobacter *MDGs.

### *Campylobacter* isolation and short-read sequencing

#### Microbiological culture

To validate the quality of *Campylobacter* MDGs from direct sequencing, each stool sample positive for *Campylobacter* by PCR underwent *Campylobacter* isolation by direct culture using a modified ISO method (EN ISO 10272–2019) for detecting and enumerating *Campylobacter* [49]. In brief, a 10 µl aliquot of raw stool was directly plated onto a modified charcoal-cefoperazone deoxycholate agar (mCCDA) supplemented with cefoperazone and amphotericin-B supplements (Oxoid). Additionally, for the subset of filtered stool samples, a 10 µl volume of filtrate was inoculated onto a full mCCDA plate. All plates throughout the isolation protocol were incubated in a microaerophilic atmosphere using anaerobic jars with CampyGen 2.5 litre sachet (Oxoid) at 37 °C for 48 h. *C. jejuni* strain 81116 [50] was used as a positive control throughout the protocol. Once incubated, up to 30 suspected *Campylobacter* colonies per sample were sub-cultured onto a second mCCDA for purification, incubated and further sub-cultured onto Columbia blood agar with 5% horse blood (Oxoid). Typical *Campylobacter* colony morphology, microscopy and oxidase testing (Thermo Fisher Scientific) were used on presumptive *Campylobacter* isolates. A sample was classified as *Campylobacter* positive if one or more presumptive isolates were confirmed as *Campylobacter* by sequence taxonomic profiling. Sixty-nine *Campylobacter* genomes derived from culture, which we have previously published [51], were also included in this study as part of the culture for MDG comparison. The 69 isolates were paired with the corresponding stool metagenomes extracted in this study.

#### Isolate DNA extraction and sequencing

Presumptive *Campylobacter* isolates underwent DNA extraction using Maxwell RSC Cultured Cells DNA Kits (Promega) according to the manufacturer’s instructions. DNA libraries were prepared using an Illumina DNA Prep (Illumina), as previously described [52] and PE150 libraries were sequenced on Illumina’s Nextseq500 instrument with a mid-output flowcell (NSQ 500 Mid Output KT, v2, 300 CYS; Illumina).

#### *Campylobacter* derived from isolate genome analysis

Raw sequence reads of each sample were stored on an in-house instance of IRIDA [40]. Illumina raw reads were trimmed using fastp (v0.19.5) [42]. Analysis was performed on the trimmed reads to predict bacterial genus and species using Kraken2 (v2.1.1) [41] with the database k2_pluspf_20210517 (https://benlangmead.github.io/aws-indexes/k2). Paired-end reads were assembled using Spades (v3.12.0) [53]. The quality of the assemblies was assessed using QUAST (v5.0.2) [46]. MLST (v2.16.1) [28] was conducted using the PubMLST database (v2.16.1) [47] for *C. jejuni* to assess STs on genome assemblies. AMR determinants and gene mutations were identified using AMRFinderPlus (v3.11.4) [48].

### Statistical analysis

McNemar’s tests were used to test for differences in the sensitivity of the three detection methods (direct sequencing, qPCR and culture). For these comparisons, *Campylobacter* was considered to have been detected if it was detected in either the filtered or the unfiltered sample. Analysis was performed using RStudio (v4.1.1) [54].

### *Campylobacter* phylogenetic analysis

A subset of *Campylobacter* MDGs were selected for further phylogenetic comparison based on the results of the short-read metagenome data analysis. The metaWRAP pipeline (v1.2) [55] was used for raw sequence reads of metagenomic samples containing *Campylobacter* genomes at ≥87% BUSCO genome completeness. The pipeline is composed of further refinement of *Campylobacter* genomes using MaxBin2 [56] and Concoct [57]. MDG genomes that could not be further refined were excluded from further phylogenetic analysis. For comparisons between isolate-derived and metagenome-derived *Campylobacter* genomes, Phylonium (v1.7) [58] followed by rapidNJ (v2.3.2) [59] was used to estimate the dissimilarities between the available set of isolate-derived genomes. The Phylogenetic Diversity Analyser [60] included in IQTREE2 (v2.2.2.7) [61] was used to select the largest set of isolates that maintain phylogenetic diversity, in order to ease visualization. The newly formulated subset was merged to compare metagenome- to isolate-derived *Campylobacter* genomes of corresponding samples. For both the metaWRAP-binned MDGs and the selected isolate-derived genomes, gene extraction and annotation was conducted with Bakta (v1.8.2) [62] and a pangenome and alignment were generated with Panaroo (v1.3.3) [63]. A low threshold was set, such that any gene present in >10% of samples was included in an initial ‘core genome alignment’. Trimal (v1.4.1) [64] excluded sites with a proportion gap of >1%. This removed all genes which were not present across all sample sets and all sites with inferred insertions/deletions (indels). This approach was used to generate the final core gene alignment. IQ-TREE (v2.2.2.7) [61] software was used to build a maximum likelihood phylogenetic tree from the alignment under an HKY model [65] with gamma heterogeneity between sites [66]. ST and resistance genotype profiles were included as metadata, and were used for *in silico* metagenome- and isolate-derived genome comparisons. The Jupyter notebook [67] with analysis and visualization can be found in the GitHub repository (https://github.com/quadram-institute-bioscience/2024-campymags/tree/main/MDG_phylogeny). Additionally, an alignment-free approach was undertaken using Phylonium (v1.7) [58], directly from the refined MDGs, offering a complementary analysis to the alignment-based approach (results not shown). Both approaches were used to build phylogenetic trees containing the MDGs and culture-derived genomes recovered from the same samples.

To compare *Campylobacter* diversity between stool samples, a separate maximum likelihood phylogenetic tree using all metagenome-derived *Campylobacter* genomes meeting the inclusion criteria was built from the initial core alignment, but with trimal threshold removing sites with gaps in >10% of the samples to generate the final alignment.

### Limit of detection using long-read and *in silico* adaptive sequencing

An additional experiment, utilizing ‘background stock’ stool spiked with a known quantity of *Campylobacter*, was conducted to determine the quality of *Campylobacter* sequencing at different abundance proportions using ONT long-read sequencing on the MinION platform with two sequences setting modes: standard and adaptive. Standard sequencing refers to the conventional method where all DNA fragments are sequenced without preference, while adaptive sequencing selectively enriches for specific DNA sequences of interest during the sequencing process. One flow cell was run using standard sequencing options, while, in parallel, the second flow cell was run using adaptive sequencing with an *in silico* enrichment of 602 *Campylobacter* genus references (https://github.com/quadram-institute-bioscience/2024-campymags/tree/main/adaptive-reference).

#### Background stock metagenome DNA extraction

One stool sample with a Bristol scale rating of 5, confirmed negative for *Campylobacter* by PCR, was chosen as the background stock microbiome for this experiment. The stool sample was subjected to host DNA depletion as previously described and metagenomic DNA extraction was performed using the Maxwell RSC Fecal Microbiome DNA Kit (Promega). The resulting DNA concentration was measured in duplicate on a Qubit fluorometer using the dsDNA quantification high sensitivity kit (Life Technologies), and the final concentration of 47.6 ng µl^–1^ (in a 60 µl volume=2856 ng) was determined as the base ‘stock’ metagenome DNA.

#### *Campylobacter* DNA preparation

A previously sequenced, a clinically derived in-house *C. jejuni* isolate was re-cultured from a glycerol stock onto Columbia Blood Agar for 48 h at 37 °C under microaerophilic conditions using anaerobic jars with CampyGen 2.5 litre sachets (Oxoid). Genomic DNA from the isolate was extracted using Maxwell RSC Cultured Cells DNA Kits (Promega) following the manufacturer’s instructions, with a final concentration determined as 46.6 ng µl^–1^ by Quantiflor (Promega).

#### Stock metagenome spiking

To establish varying proportions of *C. jejuni* DNA within the stock metagenome, genomic *C. jejuni* DNA was inoculated into each stock replicate at 0.1, 0.5, 1 and 2% *Campylobacter* DNA of the total stock metagenome volume (Fig. S1). For proportions of 0.1 and 0.5%, the *C. jejuni* DNA was first diluted to 1.34 ng µl^–1^ before being added to the background stock DNA. Specifically, 2.13 µl (2.85 ng) of *C. jejuni* DNA was introduced to 60 µl of the metagenome (2856 ng) to achieve a 0.1% proportion, while 10.66 µl (14.28 ng) of *C. jejuni* DNA was added to 60 µl of the metagenome (2856 ng) for the 0.5% proportion. For proportions of 1 and 2%, *C. jejuni* DNA concentration before inoculation was 46.6 ng µl^–1^. Accordingly, 0.61 µl (28.42 ng) of *C. jejuni* DNA was added to 60 µl of the stock (2856 ng) to obtain the 1% proportion, while 1.23 µl (57.31 ng) of *C. jejuni* DNA was added to 60 µl of the stock (2,856 ng) for the 2% proportion. One replicate of stock metagenome DNA without any additional DNA from the *Campylobacter* isolate was used as a negative control to assess any potential contamination.

#### Mock microbial community standard DNA spiking

In parallel, 200 ng of a mock DNA community from ZymoBIOMICS Microbial Community DNA Standard (Cambridge Bioscience) was used [*Listeria monocytogenes* (12%), *Pseudomonas aeruginosa* (12%), *Bacillus subtilis* (12%), *Escherichia coli* (12%), *Salmonella enterica* (12%), *Lactobacillus fermentum* (12%), *Enterococcus faecalis* (12%), *Staphylococcus aureus* (12%), *Saccharomyces cerevisiae* (2%) and *Cryptococcus neoformans* (2%)]. To mimic the presence of *Campylobacter* in the mock community, 2%(4 ng) of genomic DNA from the same *C. jejuni* strain used for the stock metagenome spiking was added to the mock microbial DNA community. A qPCR assay targeting the *Campylobacter-*specific *cadF* gene was conducted to estimate the quantity of *C. jejuni* using the same assay described previously (Fig. S1).

#### Patient stool sample preparation

One aliquot of metagenomic DNA was extracted from a stool sample previously confirmed positive for *C. jejuni* by PCR, following the unfiltered stool preparation and DNA extraction described in raw stool preparation. The DNA yield was 2534 ng with a CT value of 23.83.

#### Library preparation for long-read sequencing

Using an SQK-LSK109 ligation kit (ONT) for MinION platform sequencing, metagenome DNA libraries were prepared for each sample following the manufacturer’s instructions without optional procedures or size selection. Following an equimolar pooling of barcoded samples, the same DNA library from each sample was divided into equal duplicates and loaded into two different FLO-MIN106D (R9.4.1) flow cells of MinION platforms (ONT). One flow cell was run using standard sequencing options while in parallel the second flow cell was run using an *in silico* enrichment of 602 *Campylobacter* genus references (chromosomes and plasmids) referred to as adaptive sequencing. MinKNOW (v4.5.0) software (ONT) was used to run each flowcell sequencing for 72 h.

#### Long-read metagenome data analysis

Raw sequence reads of each sample were stored on an in-house instance of IRIDA [40]. Raw data were base-called and demultiplexed using the Guppy GPU basecaller (super accuracy model, v6.0.6, ONT). Demultiplexed and base-called reads were then mapped against a database of 602 *Campylobacter* genus references containing chromosomes and plasmids using minimap2 (v2.19) [68] with default parameters. *Campylobacter* genus reads were extracted and filtered with a minimum length of 800 bp using filtlong (v0.2.0) (https://github.com/rrwick/Filtlong) and adapters were trimmed using Porechop (v0.2.4) (https://github.com/rrwick/Porechop). Filtered reads were then assembled using Flye (v2.9) [69,70] and the assembly graphs were visualized with Bandage (v0.8.1) [71] to evaluate the circularity of *C. jejuni* genomes assembled from the metagenomes. Assemblies were polished using Medaka (v1.3.2) (https://github.com/nanoporetech/medaka) and were subjected to frameshift correction using proovframe (v0.9.7) [72]. Prokka (v1.14.5) [73] was used to annotate the frameshift corrected genome assemblies, and BUSCO (v3.0.2) [44] was used to evaluate the genomes completeness. MLST and AMR determinants were identified using the PubMLST [47] and AMRFinderPlus (v3.10.16) [48] databases, respectively.

## Results

Between August 2020 and June 2022, 42 stool samples were collected from the EPA diagnostic laboratory, of which 37 were PCR+ and five were PCR- for the presence of *Campylobacter* by rapid PCR assay. Stool samples were categorized into Bristol scale values by visual assessment and ranged from 3 to 7 (Table 1). Of the 37 PCR+ and five PCR- samples, 22 and two samples respectively resulted in analysable filtrates and were included for consideration in further analysis as filtered samples.

**Table 1.** Description of 42 stool sample characteristics and detection success using rapid PCR, qPCR, culture and direct sequencing to species level

RapidPCRdetection(no. of samples)	Sample type	Bristol scale	No. of samples	Detection by culture, *n* (%)	Detection by qPCR, *n* (%)	Detection by direct sequencing, *n* (%)
PCR+ (*n=37*)	Unfiltered (*n=*37)	7	15	11 (73.33)	15 (100)	9 (60)
		6	8	7 (87.5)	8 (100)	3 (37.5)
		5	11	5 (45.45)	9 (81.81)	2 (18.18)
		4	2	2 (50.0)	2 (100)	1 (50)
		3	1	1 (100)	1 (100)	1 (100)
	Filtered subset (*n=*22)	7	7	5 (71.42)	5 (71.42)	2 (28.57)
		6	3	3 (100)	2 (66.66)	2 (60.66)
		5	9	3 (33.33)	4 (44.44)	3 (33.33)
		4	2	1 (50)	1 (50)	2 (100)
		3	1	1 (100)	1 (100)	0 (0)
	Overall PCR+ (*n=*37)		37	27 (72.97)	36 (97.30)	24 (64.86)
	Unfiltered (*n*=5)	7	1	0 (0)	0 (0)	0 (0)
PCR- (*n=5*)		6	2	0 (0)	1 (50)	0 (0)
		5	2	0 (0)	0 (0)	0 (0)
	Filtered subset (*n=*2)	6	1	0 (0)	1 (100)	0 (0)
		5	1	0 (0)	0 (0)	0 (0)
	Overall PCR- (*n=*5)		5	0 (0)	1 (20)	0 (0)

*n*: number of samples.

The overall detected proportion of *Campylobacter* was 65% (24/37) by direct sequencing, 73% (27/37) by culture and 97% (36/37) by qPCR (Table 1). There was no statistically significant difference between the sensitivity of direct vs culturing in the detection of *Campylobacter* (McNemar’s test, *P*=0.505). However, qPCR was significantly more sensitive than both (McNemar’s test *P*=0.0076 for qPCR vs culturing and *P*=0.0015 for qPCR vs direct plating). One reported PCR- stool sample contained detectable amounts of *Campylobacter* by qPCR in the unfiltered (CT=33) and filtered (CT=35) sample, but no *Campylobacter* genomes were recovered by direct whole genome sequencing (WGS) or by culture (Table 1).

### Assessment of quality metrics for *Campylobacter* MDGs

**Fig. 2.**
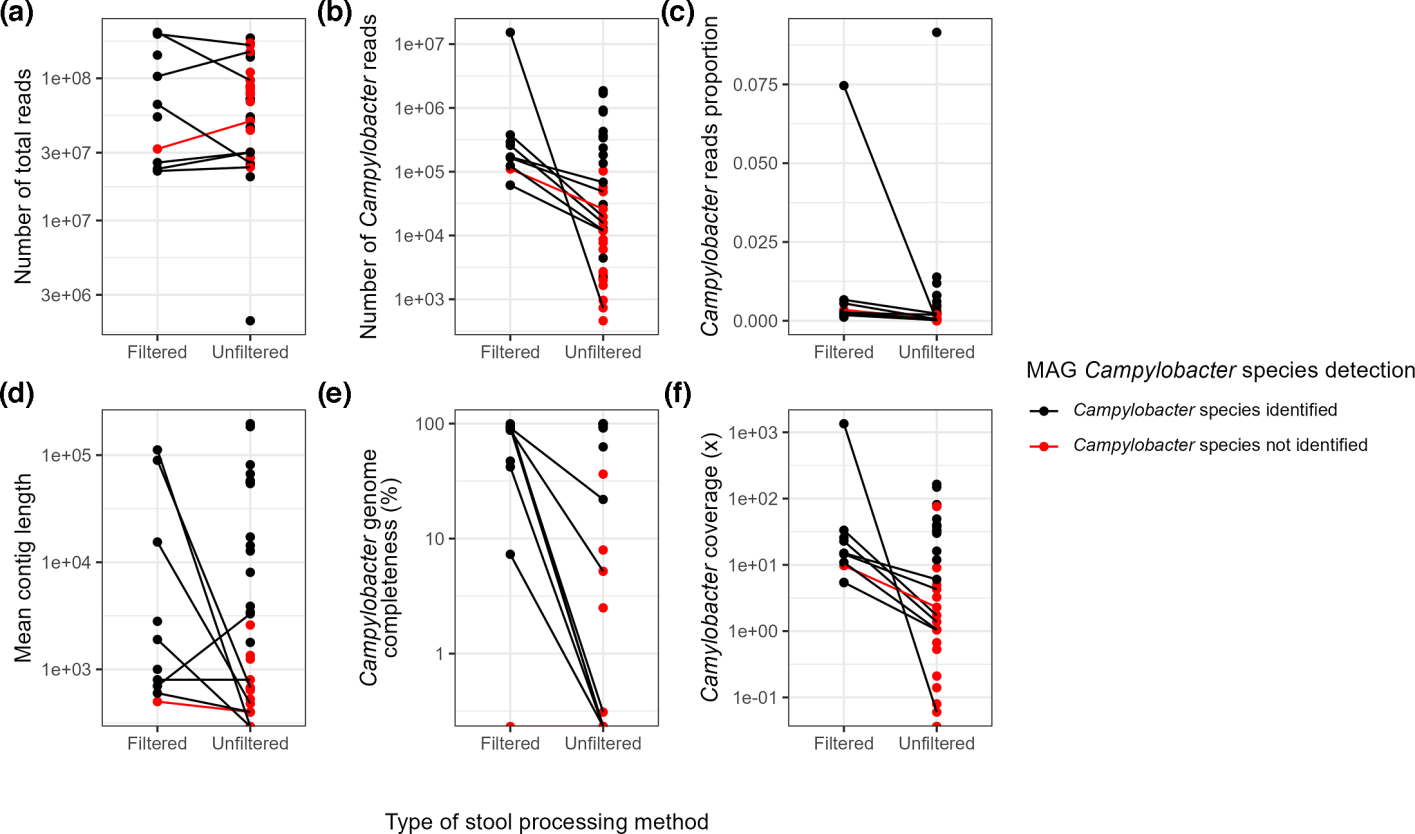
Comparison of sequence quality metrics for the characterization of *Campylobacter* to the species level from filtered and unfiltered stool samples using metagenome-derived *Campylobacter* genomes.

Of the 42 stool samples collected, 39 unfiltered samples (35 PCR+ and 4 PCR-) and 12 filtered samples (10 PCR+ and 2 PCR-) formed libraries, passed sequencing preparation quality control and were therefore included in the downstream analysis (Table S2). No PCR- sample metagenomes contained sufficient *Campylobacter* reads for MDG recovery, and therefore *Campylobacter* identification metric comparisons were conducted on 35 unfiltered PCR+ samples and 10 PCR+ filtered samples. Bioinformatic metrics to assess the quality of sequencing were the number of *Campylobacter* reads, coverage of *Campylobacter* reads, the proportion of *Campylobacter* reads in the total metagenome, genome completeness (Table 2) and genome assembly size distribution (N50) (Figs2  3). Genome completeness was used as an assessment metric for characterizing *Campylobacter* genomes to the genus, species and ST levels (Table 3). Characterization of *Campylobacter* to the species level varied and was dependent on a combination of *Campylobacter* read counts and coverage. For unfiltered samples with >12 500 *Campylobacter* reads with >5× read coverage, 94% (14/15) were characterized to the species level (Table 2) and 74% (11/15) were characterized to the ST level (Table 3) using >60% genome completeness as a metric.

**Fig. 3.**
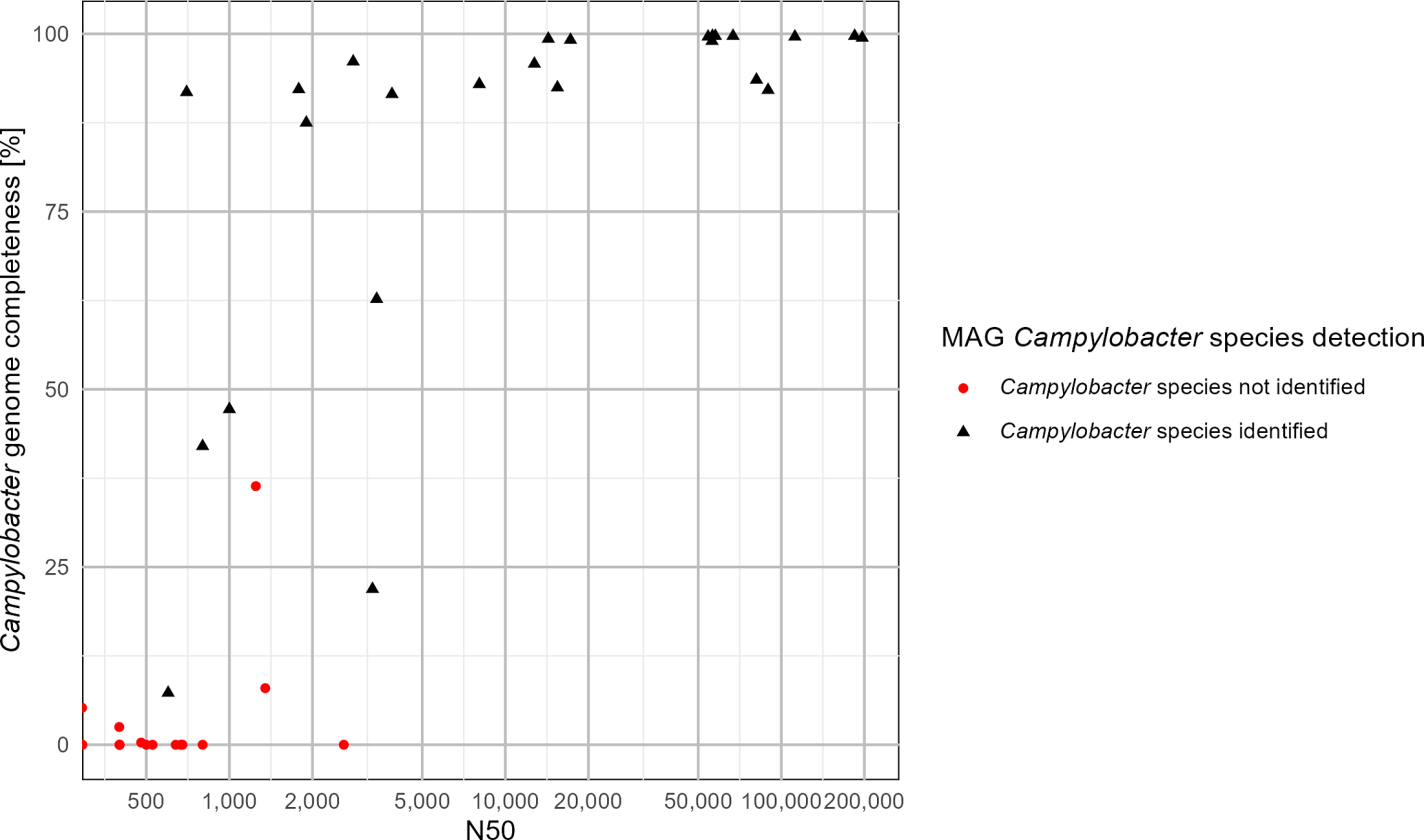
Scatterplot of 42 stool samples indicating samples for which *Campylobacter* species were identified by the percentage of *Campylobacter* genome completeness and categorized by the metagenome-derived *Campylobacter* genome size distribution (N50) metric.

**Table 2.** *Campylobacter* reads, genome completeness and proportion of *Campylobacter* detection to the species level stratified by PCR results and sample type in stool sample metagenomes passing sequencing QC by direct short-read sequencing

Rapid PCR detection	Sample type	No. of *Campylobacter* reads (*n*)	Coverage of *Campylobacter* reads (*n*)	Abundance of *Campylobacter* reads, range (%)	Genome completeness, range (%)	% of samples characterized to species level (*n*)
PCR+	Unfiltered (*n*=35)	<12 500 (*n*=15)	<5 (*n*=12)	0–0.05	0–3.26	0 (0)
			≥5 (*n*=3)	0.01–010	2.5–95.77	66.7 (2)
		≥12 500 (*n*=20)	<5 (*n*=5)	0.02–0.19	0–36.7	0 (0)
			≥5 (*n*=15)	0.01–9.15	0–99.7	93.3 (14)
	Filtered (*n*=10)	<12 500 (*n*=0)	<5 (*n*=0)	–	–	–
			≥5 (*n*=0)	–	–	–
		≥12 500 (*n*=10)	<5 (*n*=0)	–	–	–
			≥5 (*n*=10)	0.11–7.47	0–99.6	90 (9)
PCR-	Unfiltered (*n*=4)	<2500	0 (*n*=4)	–	–	–
	Filtered (*n*=2)	0–8900	0 (*n*=2)	–	–	–

*n*: number of samples; –: data insufficient to conduct analysis.

**Table 3.** Evaluation of PCR+ stool samples using *Campylobacter* genome assembly from metagenome-derived genomes for the identification of *Campylobacter* to the species and sequence type level

Rapid PCR detection	Sample type	Genome completeness, % (*n*)	% of samples characterized to species level (*n*)	% of samples characterized to sequence type (*n*)
PCR+	Unfiltered (*n*=35)	<60% (20)	5 (1)	0 (0)
		>60% (15)	100 (15)	73.33 (11)
	Filtered (*n*=10)	<60% (4)	75 (3)	0 (0)
		>60% (6)	100 (6)	66.66 (4)

1 Detection of at least one antimicrobial resistance gene; *n*: number of samples.

Among the 35 PCR+ unfiltered samples, the total number of overall reads in the metagenome ranged from 1.9 million to over 192 million and *Campylobacter* reads ranged from 460 to more than 1.8 million (Fig. 2 and Table S3). The ability to identify *Campylobacter* at the species level was again dependent on the number of *Campylobacter* reads, coverage and *Campylobacter* genome completeness (Table 2). In unfiltered samples, 70% (14/20) of samples were characterized to the *Campylobacter* species level when the number of *Campylobacter* reads exceeded ≥12 500 while 13% (2/15) could be characterized when samples contained <12 500 (Table 2). The same trend was reported for filtered samples (Table 2). When comparing coverage as a metric for quality, a higher proportion of samples were characterized to the species level at coverage values >5×, regardless of sample processing (Table 2 and Fig. 2).

Sequence type identification was not possible in samples containing *Campylobacter* genomes with <60% completeness. Although species-level identification was consistent in both unfiltered and filtered samples, ST was identified in 73% (11/15) of unfiltered samples with >60% genome completeness and 67% (4/6) of filtered samples (Table 3).

In a comparison of matched filtered and unfiltered samples (*n*=8), the number of *Campylobacter* reads, the proportion of *Campylobacter* reads in metagenomes, *Campylobacter* genome completeness and coverage metrics were consistently higher in filtered samples, indicating higher quality of genomes in filtered samples, and the ability to identify *Campylobacter* to the species level was also higher in filtered samples (Fig. 2 and Table S3). Notably, the proportion of *Campylobacter* genus reads in the total metagenomes differed between the two approaches. In unfiltered samples, the number and proportion of reads were low, ranging from 734 (<0.001 %) to 68 000 (0.02%), while in the filtered samples, the proportion was higher, ranging from 61 692 (0.01%) to >15 million (0.7%) (Table S3).

For both unfiltered and filtered samples, the higher abundance of *Campylobacter* facilitated larger contig assemblies and improved detection at the species level. Consequently, N50 values correlated with *Campylobacter* identification to the species level, resulting in an increased proportion of *Campylobacter* genus reads (Figs2  3, and Table S3). Samples with N50 values <1 000 bp had a lower percentage of that could be identified to the species level (26.67%; 4/15), with varying *Campylobacter* genome completeness (Fig. 3). In contrast, a higher proportion of samples (64%; 7/11) could be identified to the species level with N50 values ranging from 1000 to 10 000, and identification was 100% (13/13) with genome completeness approaching 100% for samples with N50 values >10 000 (Fig. 3).

### Comparison of *Campylobacter* MDGs against culture-derived genomes

Species and ST were compared in a subset of 26 PCR+ stool samples for which cultured *Campylobacter* genomes and a corresponding *Campylobacter* MDG was available. At a >60% genome completeness, the *Campylobacter* MDG identification had 100% agreement with culture-derived genomes at the species level for both unfiltered and filtered samples. Agreement at the ST level was lower with 77% of *Campylobacter* MDGs from unfiltered samples and 80% of filtered samples in agreement with culture-derived genomes (Table 4). At <60% genome completeness, only one out of 11 samples (9%) showed agreement at the species level, and no agreement was reported at the ST level.

**Table 4.** Identification of species and sequence types of metagenome-derived *Campylobacter* from PCR+ samples compared against culture-derived *Campylobacter* genomes

Metagenome sample source (*n*)	MDG-*Campylobacter* genome completeness, % (*n*)	% of MDG-*Campylobacter* sample agreement to genomes (*n*)	
		Species level	ST level
PCR+ unfiltered stool (24)	<60 (11)	9.09 (1)	0 (0)
	>60 (13)	100 (13)	76.92 (10)
PCR+ filtered stool (7)	<60 (2)	100 (2)	0 (0)
	>60 (5)	100 (5)	80 (4)

MDG: metagenome-derived genome; *n*: number of samples; ST: sequence type.

**Fig. 4.**
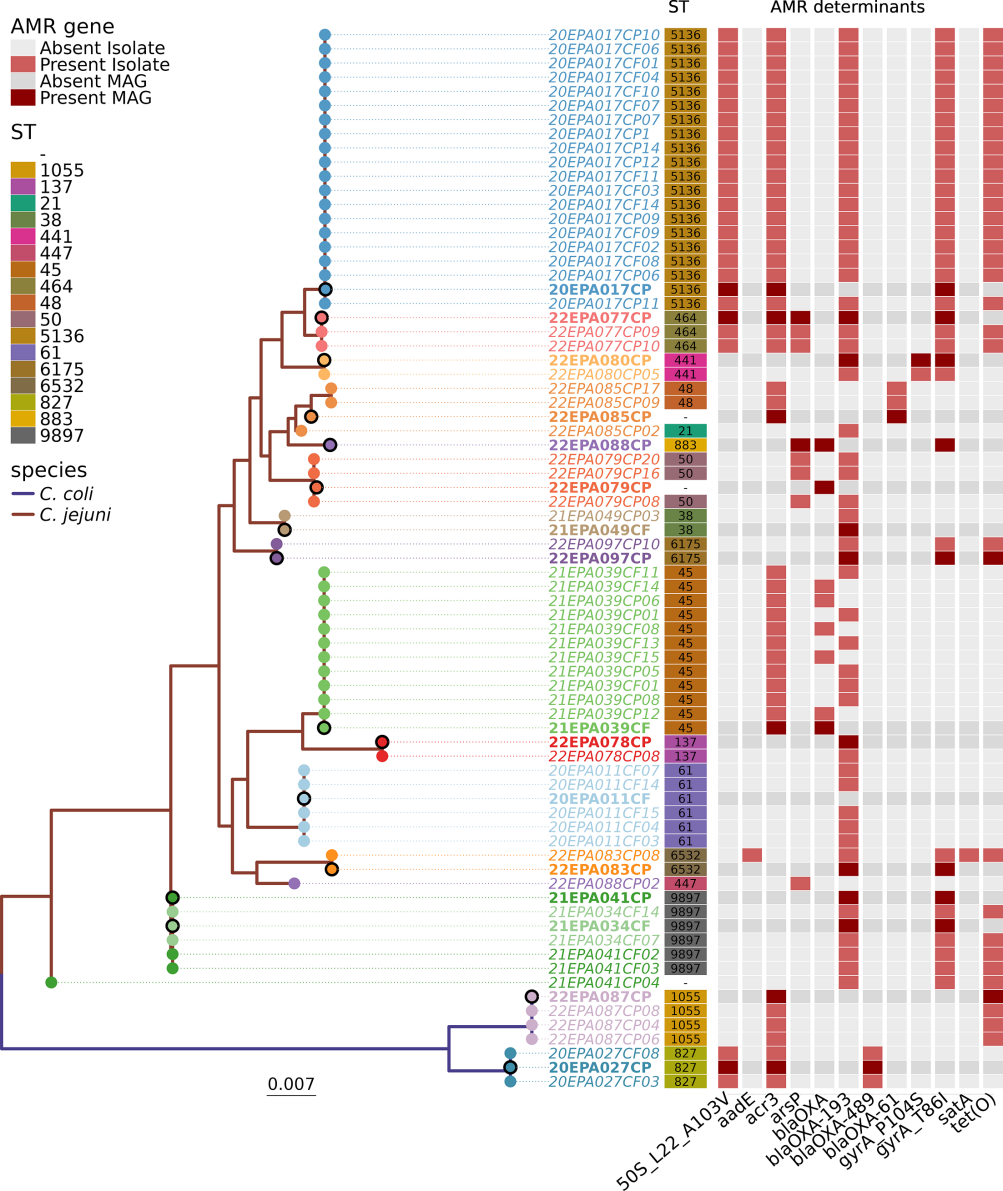
Maximum-likelihood phylogeny of *Campylobacter* strains using a core genome alignment of *Campylobacter* genomes derived from isolates (indicated by different sample ID colours) and corresponding MDG-*Campylobacter* (bold colour) from each stool sample. Legend indicates *C. jejuni* and *C. coli* species; sequence types (ST) and antimicrobial resistance determinants are indicated by the presence (red) or absence (grey) of the gene or mutation with metagenome-derived *Campylobacter* genomes indicated in bold shades.

Of the samples meeting inclusion criteria for phylogenetic comparison, 16 *Campylobacter* MDGs were compared (Fig. S2). The STs were diverse with no association of ST to sequencing success (Fig. 4). The phylogenetic relationship between culture-derived and metagenome-derived *Campylobacter* genomes from corresponding samples (paired) was measured for 14 samples containing *C. jejuni* and two samples containing *C. coli* which met the analytical inclusion criteria. The subset of samples had a diverse population of *Campylobacter* with 17 different STs represented. Of the 16 paired *Campylobacter* sample sets, 81 % (13/16) of MDGs shared the same ST with all represented isolate-derived genomes from the same sample and clustered within short branch lengths (Fig. 4). For the remaining three paired sets, sample 22EPA079 clustered with short branch lengths between culture- and metagenome-derived genomes, but the MDG *C. jejuni* ST was not identified. Sample 22EPA085 contained two different culture-derived *C. jejuni* STs and the metagenome-derived *C. jejuni* ST was not identified. Although the genomes clustered, the branch lengths were longer than the matched ST pairs. Sample 22EPA088 contained culture- and metagenome-derived *C. jejuni* of two distinct STs with no phylogenetic clustering (Fig. 4).

*In silico* resistance gene detection comparison between culture- and metagenome-derived genomes identified a high level of agreement at the antimicrobial class level for macrolides (100%; 16/16), aminoglycosides, quinolone and heavy metals (94%; 15/16), and β*-*lactams (82%; 13/16). Tetracycline-class resistance agreement was lower at 69% (11/16) (Fig. 4). At the AMR gene or mutation level, 50% (8/16) contained the same MDG and culture-derived resistance genotype profiles, 44% (7/16) of samples contained *Campylobacter* MDGs with fewer AMR determinant genes than culture-derived genomes and one *Campylobacter* MDG contained more AMR determinant genes than the culture-derived counterpart (Fig. 4).

### Limit of detection and *Campylobacter* genome enrichment during sequencing

ONT MinION sequencer standard (S) and adaptive (A) sequence settings were used to investigate the limit of detection, characterization of key clinical attributes and quality of selective read enrichment during sequencing for *Campylobacter*. In the mock community with *Campylobacter* DNA added at 2% of the total DNA volume and in the stool stock with 1 and 2% *Campylobacter* DNA added to the total DNA volume, the number of *Campylobacter* bases sequenced and genome coverage using the adaptive sequence setting was notably higher than standard sequencing (Table 5). The difference in *Campylobacter* base yield and coverage was not notable between the two settings at lower abundance (<1%). In the clinical sample (22EPA077CP) with no further *Campylobacter* DNA added the adaptive setting resulted in a 1.4× greater number of bases and notably higher coverage (Table 5). Assembly quality was low for samples with 0.5 and 0.1% of *Campylobacter* DNA, leading to increased contig counts and reduced N50 values, thereby hampering the retrieval of comprehensive genome characterization.

**Table 5.** Assembly quality and characterization of *Campylobacter* attributes in stool metagenomes spiked with varied *Campylobacter* genome levels A comparison of standard and adaptive sequencing settings on the MinION ONT sequencer platform.

Sample*+Campylobacter*%	Metagenome-derived *Campylobacter* genome quality							*Campylobacter* characterization			
	qPCR *cadF* [CT value]	Sequencer setting	No. of reads	Coverage	Genome completeness (%)	N50	No. of contigs	Species	Genome length	ST	AMR profile
Stool stock +2%	25.5	A	40 309	31	93.5	1 740 382	1	*C. jejuni*	1 740 382	2066	*tet* (O)
		S	41 947	21	92.8	1 740 377	1	*C. jejuni*	1 740 377	2066-like	*tet* (O)
Stool stock +1%	23.2	A	22 794	14	92.4	1 740 365	1	*C. jejuni*	1 740 365	2066	*tet* (O)
		S	20 884	10	92.2	1 740 389	1	*C. jejuni*	1 740 389	2066	*tet* (O)
Stool stock +0.5%	27.3	A	2721	5	51.0	36 443	39	*C. jejuni*	893 564	–	–
		S	2103	5	47.0	53 281	28	*C. jejuni*	822 742	–	–
Stool stock +0.1%	31.9	A	1115	1	–	20 451	3	*C. jejuni*	26 062	–	–
		S	1760	1	–	24 529	6	*C. jejuni*	51 408	–	–
Stool stock only	0	A	273	0	–	–	–	–	–	–	–
		S	158	0	–	–	–	–	–	–	–
Mock community only	0	A	436	0	–	–	–	–	–	–	–
		S	143	0	–	–	–	–	–	–	–
Mock community +2%	20.9	A	378 862	54	92.7	1 740 426	1	*C. jejuni*	1 740 426	2066	*tet* (O)
		S	110 142	17	93.6	1 740 426	1	*C. jejuni*	1 740 426	2066	*tet* (O)
In-house *C. jejuni* isolate (20EPA012CF09)	–	S	13 616	51	–	1739709	1	*C. jejuni*	1 740 253	2066	*tet* (O)
Clinical sample metagenome (22EPA077CP)	23.8	A	43 944	7	93.5	1 643 393	21	*C. jejuni*	1 791 826	464	*arsP*; *acr3*; *bla*_OXA-193_; *tet* (O)
		S	27 267	5	93.2	259 082	15	*C. jejuni*	1 763 594	464-like	*arsP*; *acr3*, *bla*_OXA-193_; *aadE*; *tet* (O)
Clinical sample isolate genome (22EPA077CP)	–	S	40 123	67	96.92	1 081 145	4	*C. jejuni*	1 783 848	464	*arsP*; *acr3*; *bla*_OXA-193_; *50S+L22_A103V*; *gyrA* (T86I); *tet* (O)

CT: cycle threshold; A: Adaptive setting on the ONT sequencer; MAG: metagenome-assembled genome; N50: assembly size distribution; S: Standard setting on the ONT sequencer; ST: sequence type; AMR: antimicrobial resistance; -–: not detected/data insufficient for analysis.

In our study, when evaluating genome completeness using BUSCO (Table S5), adaptive sequencing generally showed a slight improvement in completeness metrics compared to standard settings, especially at higher *Campylobacter* concentrations (Table 5). Both methods reached a limit of reliable detection and characterization at lower *Campylobacter* DNA concentrations.

There was agreement of ST and AMR genotype profile characterization of metagenome-derived and culture-derived genomes at 2 and 1% *Campylobacter* DNA added, but characterization of ST and AMR genotypes could not be made for lower abundance samples (Table 5). For the clinical sample, characterization could be made at the ST level, but there was inconsistency when comparing AMR genotype profiles (Table 5).

**Fig. 5.**
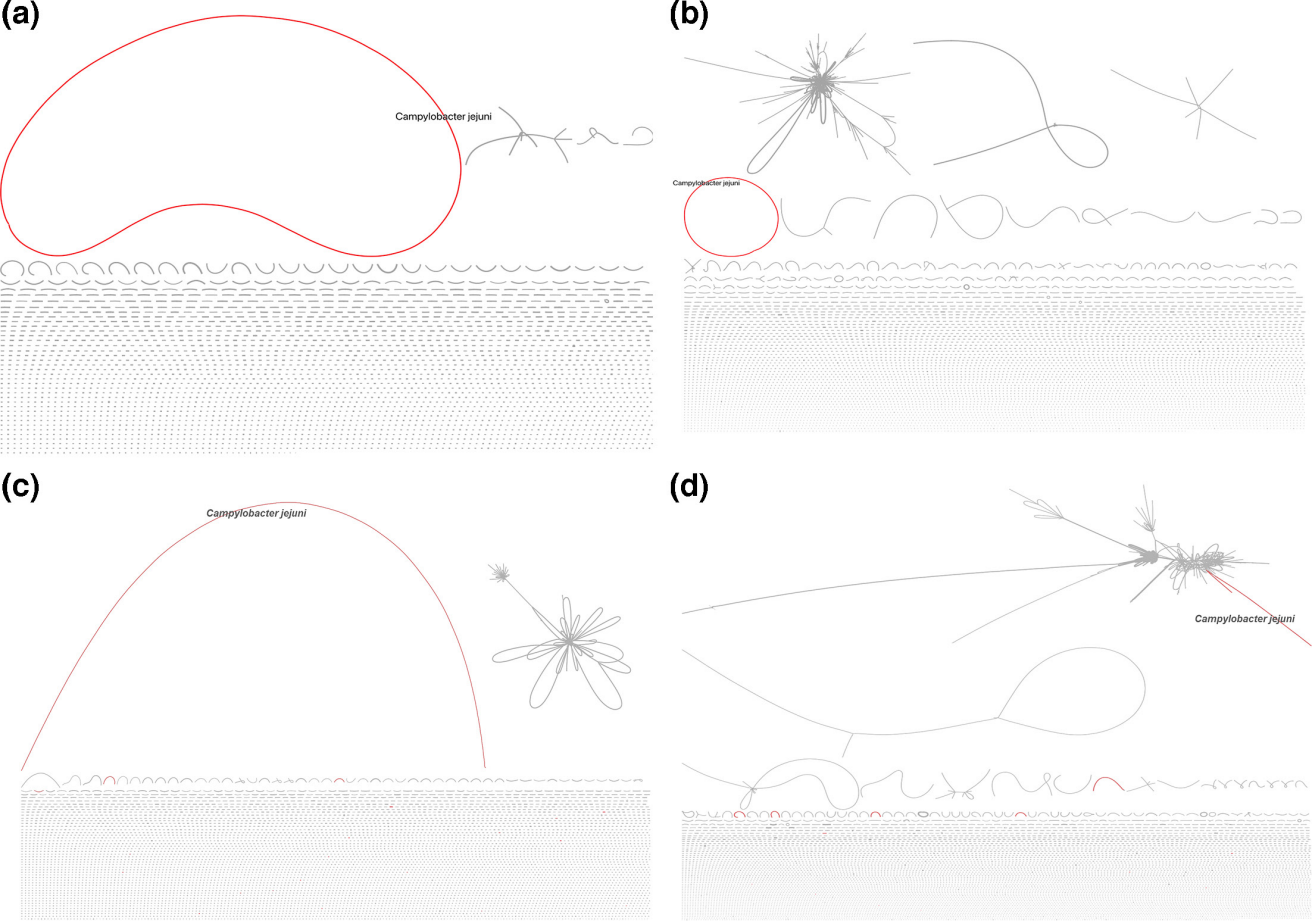
Comparison of *Campylobacter* genome assemblies from standard and adaptive ONT sequencing: visualization of genome circularity. The four subfigures illustrate the circularity of the assembled metagenome-derived *Campylobacter* genomes under different conditions. *Campylobacter* genomes are shown in red using Bandage software, with ring size relative to other assemblies within the sample. (**a**) Stock stool metagenome with 2% *Campylobacter* DNA using adaptive sequencing. (**b**) Stock stool metagenome with 2% *Campylobacter* DNA using standard sequencing. (**c**) An unknown abundance of *C. jejuni* in a clinical stool metagenome, prepared using the Adaptive sequencing setting. (**d**) The same clinical stool metagenome as in (c) sequenced using the Standard sequencing setting.

When examining genome assemblies of *Campylobacter* obtained from stool samples and mock microbial communities using standard and adaptive nanopore sequencing methods, we observed variations in genome circularity between the two sequencing approaches. Specifically, adaptive sequencing consistently resulted in assemblies with higher circularity compared to the standard setting sequencing across all tested conditions (Figs 5 and S3).

## Discussion

This study establishes how precision-based metagenomic metrics can be used to determine the downstream quality of clinically important *Campylobacter* genome attributes and how direct sequencing compares to the ‘gold standard’ of culture. Clinical stool samples originating from gastroenteritis patients vary in structure, volume and composition, providing challenges to testing and pathogen characterization tools [74]. In this study of samples with diverse Bristol scales, the success of identification beyond the presence/absence of detection of the genus to species level was highly dependent on sample input – the initial material or sample used for analysis, encompassing factors such as quantity, quality and any pre-processing steps applied before analysis. Additionally, the quantity of *Campylobacter* reads obtained during sequencing and the quality of sequenced products influenced the accuracy and depth of species-level identification. qPCR proved to identify species significantly better than culture or direct sequencing, but the limitation of *Campylobacter* attribute information does not allow for the technique to be exclusively used for clinical and epidemiological relevance. Culture, considered to be the gold standard for genome-level characterization, is expensive, laborious and time-consuming yet to this point a suitable alternative solution for precise genome-level characterization to ST and AMR profiling has not been demonstrated.

Detection of low-abundance pathogens in clinical stool samples through metagenomic methods is difficult [20]. Previous clinical metagenome studies have validated the detection at the genus level but did not provide further clinically relevant characterization [75]. This study validated that direct sequencing was equivalent to culture in identifying *Campylobacter* STs, and the high agreement of matched culture- and metagenome-derived *Campylobacter* genomes at high depth of coverage and genome completeness validated the tools and metrics needed for reliable application of direct sequencing into clinical and epidemiological surveillance settings. The genome characteristic from direct sequences was supported by clinical metagenome studies testing bioinformatic pipelines for accuracy and demonstrated similar results to previous metagenome-based studies [20,21, 76]. Metagenome accuracy of species-level identification revealed that *Campylobacter* spp. detection rates are dependent on factors such as read count, coverage and genome completeness, as highlighted in the literature and previous studies [32, 77,78].

Our findings align with the consensus that the completeness of genomes significantly influences the accuracy and reliability of such characterizations. Previous studieshave established a benchmark, suggesting that a bacterial MDG should ideally exhibit a CheckM completeness of more than 85% to be considered acceptable. However, whilst it is important to acknowledge that the field recognizes the practicality of using MDGs with completeness values as low as 70% for functional analyses of microbial communities, it is also essential to consider the context. For individual or surveillance purposes, where precision and reliability are paramount, higher completeness values may be necessary. However, for large-scale research studies, where the emphasis is on broader trends and patterns rather than individual-level precision, a completeness threshold of 70% may be deemed acceptable. This difference in standards could be likened to the distinction between research-quality and clinical-quality physiological testing, where the former prioritizes generalizability and the latter emphasizes individual diagnostic accuracy. Nevertheless, our research reaffirms the potential limitations associated with such lower completeness values.

When MDG completeness falls below 60%, the success rate for characterizing this bacterium to the species level and ST diminishes. This echoes the concerns regarding the underestimation of functional capacity in genomes with completeness values of 70% or lower [32]. Our study also aligns with the broader consensus that genome completeness is a pivotal factor in microbial characterization using MDGs [32]. However, other studies [77,79] have considered a higher threshold for completeness (>90%), and this criterion was defined by the minimum information obtained from MDGs. Nevertheless, the minimum completeness threshold for a genome to be considered high-quality can vary depending on the study.

It is important to note that while our study provides completeness and contamination metrics, which are crucial for evaluating metagenomic-derived assemblies, we did not incorporate the Minimum Information about a Metagenome-Assembled Genome (MIMAGs) criteria regarding rRNA and tRNA gene counts [77]. Including these criteria in future analyses could offer a more comprehensive assessment of assembly quality and better align with MIMAG standards. This may provide further validation within the established metagenomic assembly guidelines.

The accuracy of resistance genotype profiles and lineage clusters between metagenome- and culture-derived genomes at the class level in this study has not previously been reported for *Campylobacter*. Our study reveals that higher genome completeness, especially when exceeding 60%, significantly increases the likelihood of detecting AMR genes. This observation aligns with previous research [78], which emphasizes the challenges posed by organismal complexity, contamination, low read depth and strain heterogeneity in achieving complete genomes for characterization.

Antimicrobial use for campylobacteriosis is not the first line of treatment options, but when required fluoroquinolones and macrolides are provided by clinical guidelines as suitable options [80]. Our metagenome-derived results had high agreement at the macrolide (100%) and fluoroquinolone (94%) levels with culture isolates, supporting our metagenomic pipeline as a tool for resistance profiles of *Campylobacter* directly through stool.

The N50 threshold also proved to be an important factor influencing the quality of MDGs for *Campylobacter* species detection. This observation in our study aligns with the broader implications discussed in previous studies [81,82]. Our findings resonate with the idea that the N50 threshold has substantial consequences for the quality and completeness of MDGs [77]. In our case, this was evident in the 100% *Campylobacter* detection rate achieved in samples with N50 values exceeding 10 000. In the context of *Campylobacter* species detection, our study echoes the concept that the reliability of MDGs in representing natural populations can be affected by the N50 threshold. For example, in a study [18] that used a 10 kb threshold for contig length to ensure high-quality MDGs, the reliability in representing natural populations was enhanced. Similarly, in our study, we observed a direct correlation between N50 values and *Campylobacter* detection reliability, which reinforces the idea that a higher N50 threshold contributes to more trustworthy genomes derived from metagenomes, thereby reflecting the true diversity and composition of microbial populations in the studied samples.

In this study, some samples with lower read counts still achieved high MDG completeness, suggesting that it was compensated with other sequencing quality metrics such as higher coverage and higher N50, and one metric alone cannot be used as a sole guide to quality. A previously reported study concluded that even at a lower read count, if the sample coverage is comprehensive, MDG assembly is possible and the assembly process becomes more robust as coverage rises, increasing the recovery of essential genomic information accurately [83,84].

It is more common to construct phylogenetic trees of *Campylobacter* species based on whole-genome sequences derived from cultured isolates. We successfully demonstrated the phylogenetic relationship between culture-derived and metagenome-derived *Campylobacter* genomes from corresponding samples. This indicated the reliability of our culture-free approach as 82% (13/16) of MDGs shared the same ST with all represented isolate-derived genomes from the same sample.

Some limitations in phylogenetic analysis persist and are a challenge to using metagenomics for samples with low completeness scores and samples with mixed *Campylobacter* strains. For instance, the pair from sample 22EPA079 clustered with short branch lengths, whereas the MAG *C. jejuni* ST was not identified as this sample had low genome completeness (63%). This resonates with the fact that low genome completeness in *Campylobacter* can compromise accuracy in identifying genetic markers essential for characterization [83,84].

In the phylogenetic analysis of this study, we used a low inclusion threshold into the initial ‘core gene set’ to allow for all genes present even in a small fraction of the samples to bin the genome-level alignment. This alignment can then be further refined depending on the subset of samples of interest.

In mixed strain samples containing two different culture-derived *C. jejuni* STs the pipeline could not separate reads from *Campylobacter* MAGs into two different STs. Co-infections with multiple *Campylobacter* species, while less common than single-species infections, have been reported with varying prevalence rates, typically ranging from 7 to 7.5% in human clinical samples [85]. The presence of co-infections can complicate both genome assembly and sequence typing due to the mixed genetic material from different species or strains. This can result in several challenges [85,86]. In genome assembly, the intermingling of reads from different *Campylobacter* species can lead to chimeric contigs or scaffolds, where sequences from different species are incorrectly joined together, complicating downstream analyses, and reducing the overall quality and accuracy of the assembly [87,89]. When assembling genomes from samples containing multiple *Campylobacter* species, there is a risk of creating chimeric contigs where sequences from different species incorrectly join together [90]. This can occur due to the similarity between *Campylobacter* genomes since distinguishing between closely related strains or species during the assembly process is difficult due to the assembly algorithms’ struggle to correctly separate and reconstruct the genomes of individual species when working with mixed sequence data [91].

For ST detection, co-infections can result in the detection of mixed STs, making it difficult to assign a clear ST to the sample [92]. This ambiguity arises because sequence typing methods rely on identifying specific alleles at defined loci, and the presence of multiple alleles from different species can lead to inconclusive or mixed results [92]. There is also a risk of misidentifying the predominant ST if one species or strain is more abundant in the sample, potentially leading to incorrect epidemiological conclusions [92].

Considering these potential issues, there are still limitations to the methods, and therefore additional validation steps are needed when considering cases. Furthermore, horizontal/lateral gene transfer (HGT) and homologous recombination are recognized as dynamic processes within the genus *Campylobacter*, contributing to genetic diversity and potentially influencing clinical and public health applications [93]. This can complicate genomic analyses, particularly in metagenomic studies aimed at assessing sequence quality metrics for clinical purposes. Our study evaluated *Campylobacter* sequence quality metrics through metagenome analysis and validated the accuracy of MDGs against genomes cultured from the same stool samples. The successful demonstration of a phylogenetic relationship between culture-derived and metagenome-derived *Campylobacter* genomes, with 82% of MDGs sharing the same ST as isolate-derived genomes, highlights the reliability of our approach for clinically relevant attributes. This validation is crucial for ensuring the accuracy of genomic data interpretation in clinical and public health contexts, despite the potential influences of HGT. Moving forwards, refining methodologies to account for HGT dynamics will be essential for further enhancing the precision of genomic analyses in epidemiological studies and clinical diagnostics [93,94].

We investigated the impact of stool filtration on the recovery of *Campylobacter* MDGs, as another methodological enhancement of metagenomic analysis. Our findings align with the principles of selective filtration techniques in metagenomics as discussed in previous studies [95,98]. We observed that this selective filtration had a positive effect on *Campylobacter* genome completeness in the subset of samples that were tested. This suggests that the efficient separation of bacterial cells from stool sample debris through filtration probably contributed to the improved genome completeness observed in our filtered samples. Moreover, our findings highlight the utility of filtration in enriching the target fraction of *Campylobacter* cells in the sample. In our study, filtration improved the precision and depth of detection for ST and AMR genes compared to unfiltered samples. However, the current method is labour-intensive, raising concerns about its sustainability. Further investigation into filtration techniques is warranted to explore modifications or alternatives that balance accuracy while reducing resource demands, ensuring its feasibility for future research.

In a separate investigation in this study, we examined the impact of ONT MinION selective sequencing settings on the limit of detection and data quality, particularly within metagenomic samples spiked with varying percentages of the *C. jejuni* genome. These findings resonate with previous studies that have used MinION adaptive sequencing for diverse metagenomic applications, such as species enrichment, pathogen surveillance and clinical diagnostics [99,103]. Notably, our study revealed that adaptive sequencing settings, especially when applied to metagenomes spiked at higher abundance (1–2%), yielded robust assembly quality, characterized by high N50 values and precise ST identification. This aligns with the concept that adaptive sampling has the potential to enrich the sequencing of species of interest in metagenomic samples. Furthermore, adaptive settings increased read yield, with adaptive sequencing providing a 1.5× and 1.4× improvement in read yield compared to standard settings. This parallels with the advantages of MinION adaptive sequencing previously reported [100] where it was noted that nanopore sequencing can yield long-read sequencing data without the need for clonal library amplification, contributing to the generation of abundant data. However, with lower percentages of *C. jejuni* genome spike (0.5 and 0.1%), assembly quality started to deteriorate, leading to increased contig counts and reduced N50 values. These challenges align with the observations in a previous study [104], which emphasized the effectiveness of adaptive sequencing in enriching low-abundance DNA, but also recognized the need for optimization in challenging scenarios.

The comparison between adaptive and standard sequencing settings in real sample analysis yielded variability in assembly quality and ST assignment. Standard sequencing settings displayed slightly lower quality results. However, adaptive setting sequencing yielded 1.4× more *C. jejuni* reads compared to standard settings, reflecting its potential advantages in capturing target sequences. Using the standard settings, the MLST analysis was able to fully identify only six out of the seven housekeeping genes, whereas with the adaptive settings, all seven housekeeping genes were fully identified, enabling precise determination of the ST. This demonstrates the enhanced resolution and effectiveness of the adaptive sampling approach. Nevertheless, it is crucial to acknowledge that the MDG of this sample did not yield a complete circular *C. jejuni* genome. This highlights the complexity of stool sample composition and the need for further refinement of techniques. In our study, we observed that spiking stool samples with 2 and 1% *C*. *jejuni* DNA, as well as spiking the mock community with 2%, resulted in good genome completeness and visualized genome circularity. However, adaptive sequencing consistently produced better quality in terms of genome circularity.

The adaptive sampling method shows promise, but its implementation in metagenomic analyses, especially from complex samples such as stool, requires further optimization. Addressing these challenges will enhance the overall quality of the assembly and improve the ability of the methodology to distinguish STs more reliably, thereby maximizing the potential of this novel enrichment approach.

In our study, when the genome completeness was evaluated using BUSCO, we demonstrated that adaptive sequencing settings generally provided a slight improvement in completeness metrics compared to standard settings, particularly at higher *Campylobacter* concentrations. However, both methods face challenges at lower concentrations, suggesting a need for further refinement in sequencing and assembly techniques for samples with low pathogen abundance. While our approach did not specifically include the analysis of rRNA and tRNA genes as per MIMAG standards, the use of BUSCO provides a robust assessment of genome completeness. Future studies should consider incorporating these additional metrics to fully comply with MIMAG standards [77], offering a more comprehensive evaluation of assembly quality.

## Conclusion

This study employed a comprehensive approach to investigate *Campylobacter* detection and characterization in clinical stool samples using direct metagenomic sequencing. Our findings have highlighted the critical influence of various factors, including read count, coverage, genome completeness and genome assembly size distribution, on the accuracy and reliability of *Campylobacter* species detection and characterization of clinically relevant attributes. We have demonstrated that higher genome completeness, read counts and coverage are essential for robust species-level detection, MLST and the identification of AMR profiles. Stool filtration has proven to enhance *Campylobacter* recovery and genome completeness. Moreover, adaptive sequencing settings with ONT MinION have shown promise for improving detection and assembly quality at different abundance proportions.

The methods and insights presented in this study provide valuable guidance for epidemiologists and clinicians working with metagenomic data for *Campylobacter* detection and characterization. By optimizing sequencing and data analysis strategies, it is possible to achieve more accurate and comprehensive insights into *Campylobacter* characteristics in complex clinical stool samples without the need for culture. We have demonstrated how direct sequencing can fill the growing data gap of *Campylobacter* genomic knowledge in which laboratories can no longer provide clinical attributes from rapid PCR assays. This research contributes to the ongoing efforts to better understand and combat *Campylobacter*-related infections, which have significant public health implications.

## supplementary material

10.1099/mgen.0.001284 Uncited Supplementary Material 1.

## Abbreviations

AMR: antimicrobial resistance
*bla*_OXA_: beta-lactamase oxacillinase gene
CBA: Columbia blood agar
EPA: Eastern Pathology Laboratory
*gyrA*: DNA gyrase subunit A
HGT: horizontal gene transfer
HTA: Health Technology Assessment
ISO: International Organization for Standardization
mCCDA: modified charcoal cefoperazone deoxycholate agar
MDG: metagenome-derived genome
MLST: multilocus sequence typing
ONT: Oxford Nanopore Technologies
QC: quality control
qPCR: quantitative PCR
ST: sequence type
*tet*: tetracycline gene
WGS: whole genome sequencing
