# Supplementary material for: Capturing clinically relevant Campylobacter attributes through direct whole genome sequencing of stool
Source: Microb Genom. 2024 Aug 30;10(8):001284. doi: 10.1099/mgen.0.001284 (PMC11570993; doi:10.1099/mgen.0.001284)
Supplement: Uncited Supplementary Material 1. [file mgen-10-01284-s001.pdf]

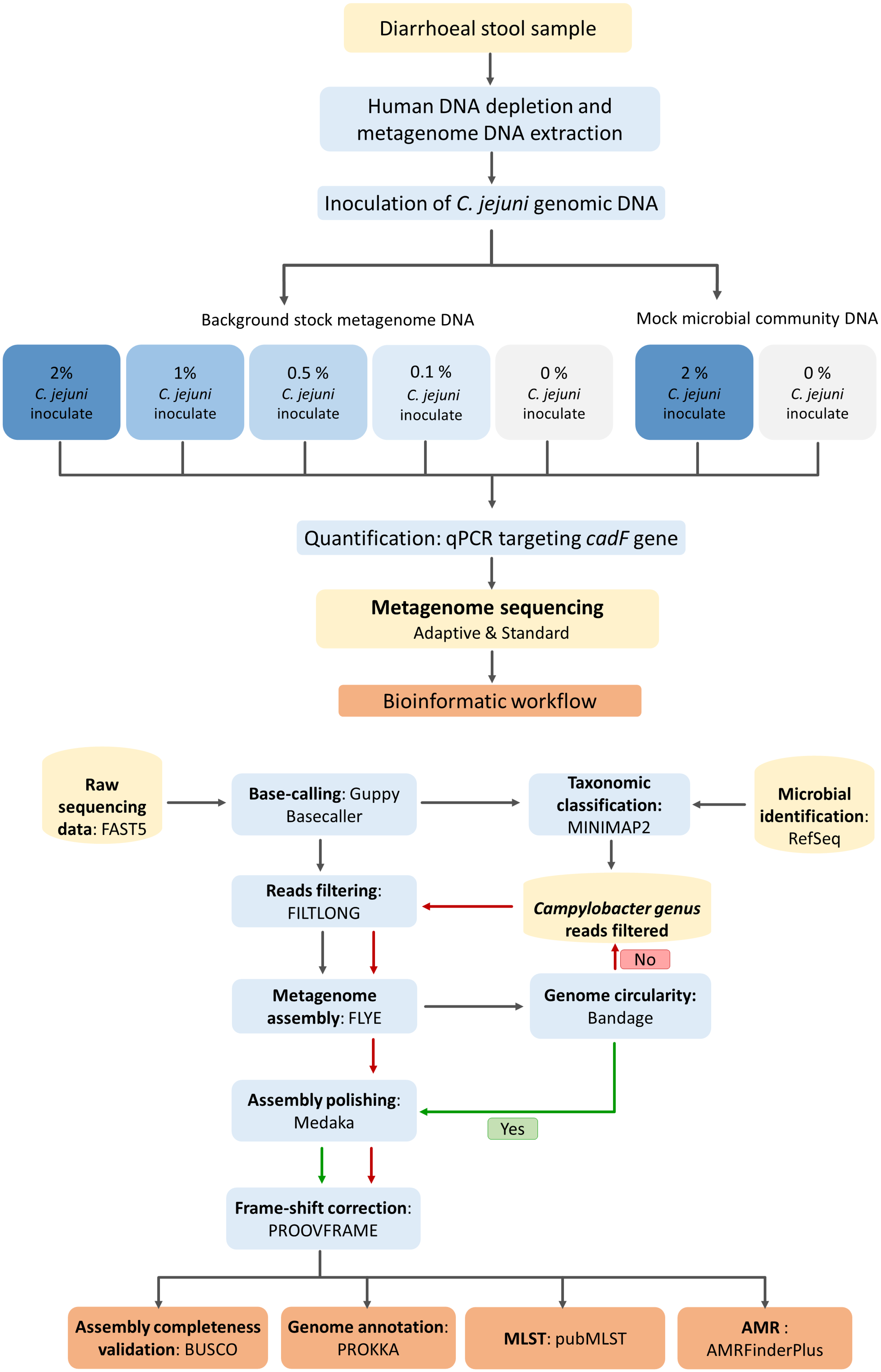

Figure S1. Experimental workflow of stool samples with varied *Campylobacter* concentration inputs for sequencing on the ONT MinION sequencer using standard and adaptive sequence setting modes, and bioinformatic pipeline for in silico analysis.

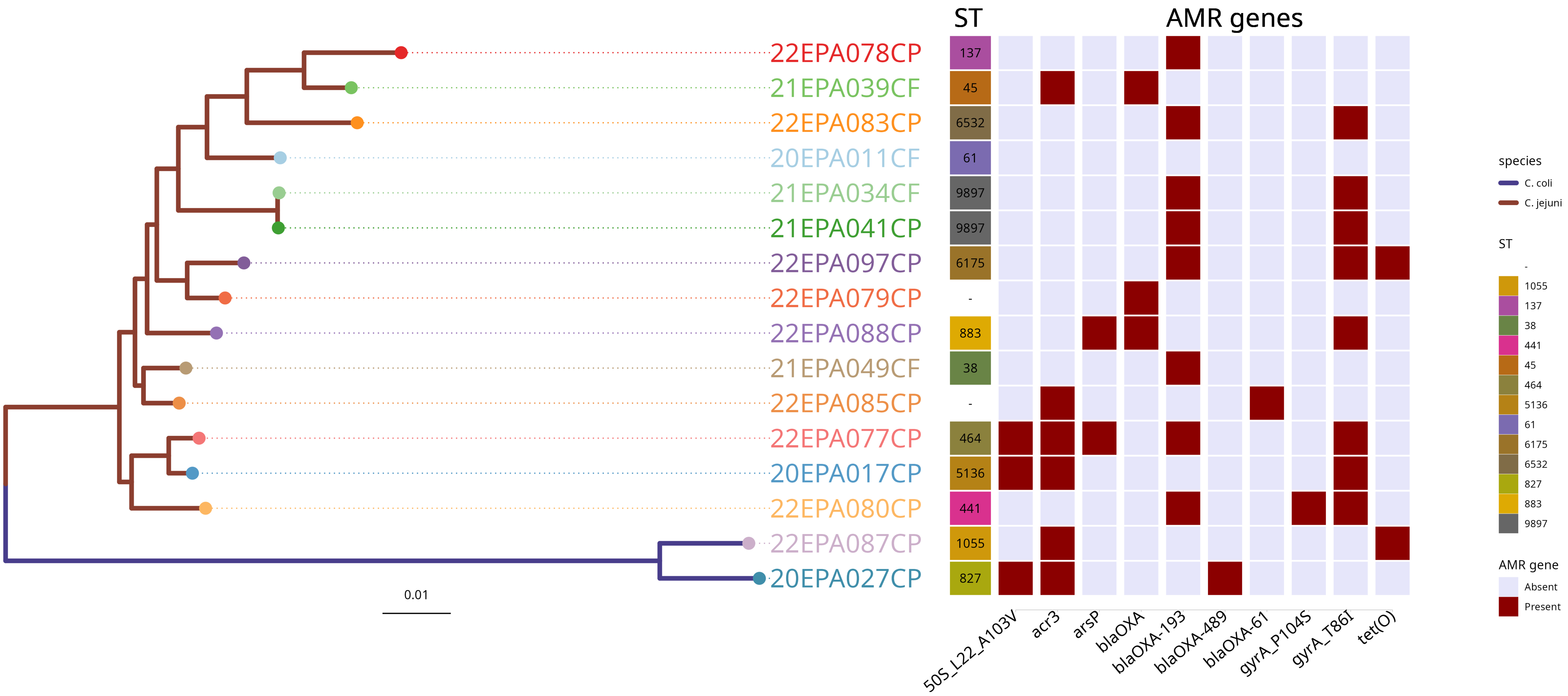

Figure S2. Maximum-likelihood phylogeny of metagenome-derived genomes of *Campylobacter* strains using core genome alignment originating from 16 stool samples. Species are indicated by blue (*C. coli*) or red (*C. jejuni*); sequence types are colour coded; antimicrobial resistance determinant genes or mutations are indicated by presence (red) or absence (grey).

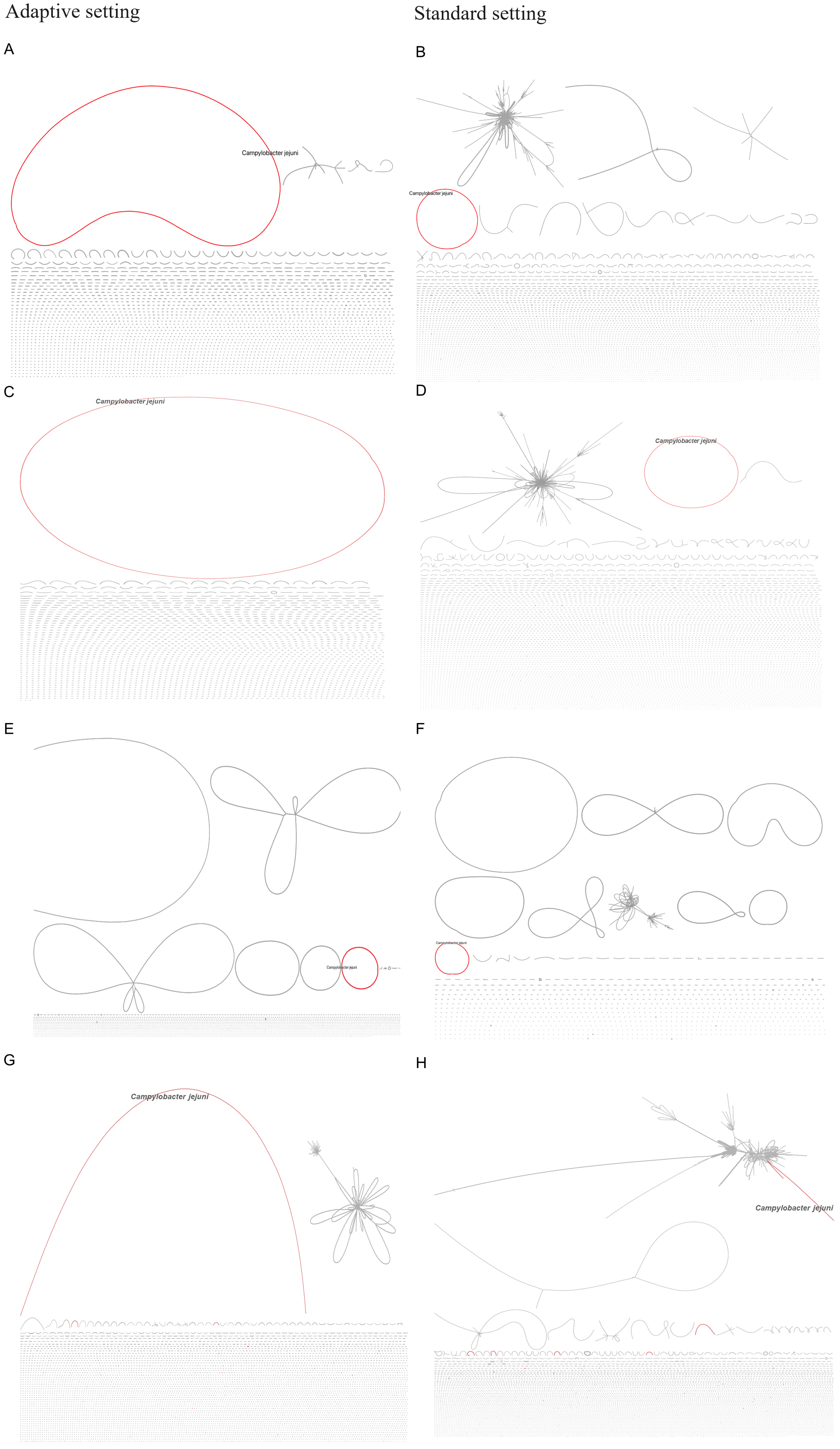

Figure S3. Visualisation of metagenome-derived *Campylobacter* genome assemblies using standard and adaptive sequencing modes on the ONT MinION sequencer. *Campylobacter* genome is indicated in red using Bandage software, with ring size relative to other assemblies within the sample. **(A)** Stock stool metagenome with 2% *Campylobacter* DNA using adaptive sequencing. **(B)** Stock stool metagenome with 2% *Campylobacter* DNA using standard sequencing. **(C)** Stock stool metagenome with 1% *Campylobacter* DNA using adaptive sequencing. **(D)** Stock stool metagenome with 1% *Campylobacter* DNA using standard sequencing. **(E)** Mock microbial community with 2% *Campylobacter* DNA using adaptive sequencing. **(F)** Mock microbial community with 2% *Campylobacter* DNA using standard sequencing. **(G)** An unknown abundance of *C. jejuni* in a clinical stool metagenome, prepared using adaptive sequencing setting. **(H)** The same clinical stool metagenome as 'G' was sequenced using the standard sequencing setting.

Table S1. Sequence Read Archive accession numbers for stool metagenomes of human gastroenteritis samples

Sample Name	BioProject	SRA accession number	Genome origin	Source
20EPA011CF	PRJNA1046283	SRR27129268	Metagenome	Human stool
20EPA013CP	PRJNA1046283	SRR27129267	Metagenome	Human stool
20EPA014CP	PRJNA1046283	SRR27129256	Metagenome	Human stool
20EPA015CP	PRJNA1046283	SRR27129245	Metagenome	Human stool
20EPA017CP	PRJNA1046283	SRR27129234	Metagenome	Human stool
20EPA026CP	PRJNA1046283	SRR27129223	Metagenome	Human stool
20EPA027CP	PRJNA1046283	SRR27129221	Metagenome	Human stool
20EPA028CP	PRJNA1046283	SRR27129220	Metagenome	Human stool
21EPA034CF	PRJNA1046283	SRR27129219	Metagenome	Human stool
21EPA034CP	PRJNA1046283	SRR27129266	Metagenome	Human stool
21EPA035CP	PRJNA1046283	SRR27129265	Metagenome	Human stool
21EPA036CP	PRJNA1046283	SRR27129264	Metagenome	Human stool
21EPA037CP	PRJNA1046283	SRR27129263	Metagenome	Human stool
21EPA039CF	PRJNA1046283	SRR27129261	Metagenome	Human stool
21EPA039CP	PRJNA1046283	SRR27129260	Metagenome	Human stool
21EPA041CP	PRJNA1046283	SRR27129259	Metagenome	Human stool
21EPA042CP	PRJNA1046283	SRR27129258	Metagenome	Human stool
21EPA048CF	PRJNA1046283	SRR27129257	Metagenome	Human stool
21EPA048CP	PRJNA1046283	SRR27129255	Metagenome	Human stool
21EPA049CF	PRJNA1046283	SRR27129254	Metagenome	Human stool
21EPA049CP	PRJNA1046283	SRR27129253	Metagenome	Human stool
21EPA050CF	PRJNA1046283	SRR27129252	Metagenome	Human stool
21EPA050CP	PRJNA1046283	SRR27129251	Metagenome	Human stool
21EPA051CF	PRJNA1046283	SRR27129250	Metagenome	Human stool
21EPA051CP	PRJNA1046283	SRR27129249	Metagenome	Human stool
21EPA052CF	PRJNA1046283	SRR27129248	Metagenome	Human stool
21EPA054CF	PRJNA1046283	SRR27129247	Metagenome	Human stool
21EPA054CP	PRJNA1046283	SRR27129246	Metagenome	Human stool
21EPA055CF	PRJNA1046283	SRR27129244	Metagenome	Human stool
21EPA055CP	PRJNA1046283	SRR27129243	Metagenome	Human stool

22EPA078CP	PRJNA1046283	SRR27129237	Metagenome	Human stool
22EPA079CP	PRJNA1046283	SRR27129236	Metagenome	Human stool
22EPA080CP	PRJNA1046283	SRR27129235	Metagenome	Human stool
22EPA083CP	PRJNA1046283	SRR27129232	Metagenome	Human stool
22EPA070CP	PRJNA1046283	SRR27129239	Metagenome	Human stool
22EPA081CP	PRJNA1046283	SRR27129233	Metagenome	Human stool
22EPA085CP	PRJNA1046283	SRR27129231	Metagenome	Human stool
22EPA086CP	PRJNA1046283	SRR27129230	Metagenome	Human stool
22EPA087CP	PRJNA1046283	SRR27129229	Metagenome	Human stool
22EPA088CP	PRJNA1046283	SRR27129228	Metagenome	Human stool
22EPA089CP	PRJNA1046283	SRR27129227	Metagenome	Human stool
22EPA091CP	PRJNA1046283	SRR27129226	Metagenome	Human stool
22EPA094CP	PRJNA1046283	SRR27129225	Metagenome	Human stool
22EPA097CP	PRJNA1046283	SRR27129224	Metagenome	Human stool
22EPA077CP	PRJNA1046283	SRR27129238	Metagenome	Human stool
20EPA026NP	PRJNA1046283	SRR27129222	Metagenome	Human stool
21EPA037NF	PRJNA1046283	SRR27129262	Metagenome	Human stool
22EPA041NP	PRJNA1046283	SRR27129242	Metagenome	Human stool
22EPA061NP	PRJNA1046283	SRR27129241	Metagenome	Human stool
22EPA062NP	PRJNA1046283	SRR27129240	Metagenome	Human stool
20EPA011CF07	PRJNA797426	SRR18166198	Genome isolate	Human stool
20EPA011CF14	PRJNA797426	SRR18166191	Genome isolate	Human stool
20EPA011CF04	PRJNA797426	SRR18166201	Genome isolate	Human stool
20EPA011CF03	PRJNA797426	SRR18166202	Genome isolate	Human stool
20EPA017CF01	PRJNA1049393	SRR27110029	Genome isolate	Human stool
20EPA017CF02	PRJNA1049393	SRR27110028	Genome isolate	Human stool
20EPA017CF03	PRJNA1049393	SRR27110017	Genome isolate	Human stool
20EPA017CF04	PRJNA1049393	SRR27110006	Genome isolate	Human stool
20EPA017CF06	PRJNA1049393	SRR27109995	Genome isolate	Human stool
20EPA017CF07	PRJNA1049393	SRR27109984	Genome isolate	Human stool
20EPA017CF08	PRJNA1049393	SRR27109977	Genome isolate	Human stool
20EPA017CF09	PRJNA1049393	SRR27109976	Genome isolate	Human stool
20EPA017CF10	PRJNA1049393	SRR27109975	Genome isolate	Human stool

20EPA017CF11	PRJNA1049393	SRR27109974	Genome isolate	Human stool
20EPA017CF14	PRJNA1049393	SRR27110027	Genome isolate	Human stool
20EPA017CP01	PRJNA1049393	SRR27110026	Genome isolate	Human stool
20EPA017CP03	PRJNA1049393	SRR27110025	Genome isolate	Human stool
20EPA017CP06	PRJNA1049393	SRR27110024	Genome isolate	Human stool
20EPA017CP07	PRJNA1049393	SRR27110023	Genome isolate	Human stool
20EPA017CP09	PRJNA1049393	SRR27110022	Genome isolate	Human stool
20EPA017CP10	PRJNA1049393	SRR27110021	Genome isolate	Human stool
20EPA017CP11	PRJNA1049393	SRR27110020	Genome isolate	Human stool
20EPA017CP12	PRJNA1049393	SRR27110019	Genome isolate	Human stool
20EPA017CP14	PRJNA1049393	SRR27110018	Genome isolate	Human stool
20EPA027CF03	PRJNA1049393	SRR27110016	Genome isolate	Human stool
20EPA027CF08	PRJNA1049393	SRR27110015	Genome isolate	Human stool
21EPA034CF07	PRJNA1049393	SRR27110014	Genome isolate	Human stool
21EPA034CF14	PRJNA1049393	SRR27110013	Genome isolate	Human stool
21EPA039CF01	PRJNA1049393	SRR27110012	Genome isolate	Human stool
21EPA039CF08	PRJNA1049393	SRR27110011	Genome isolate	Human stool
21EPA039CF11	PRJNA1049393	SRR27110010	Genome isolate	Human stool
21EPA039CF13	PRJNA1049393	SRR27110009	Genome isolate	Human stool
21EPA039CF14	PRJNA1049393	SRR27110008	Genome isolate	Human stool
21EPA039CF15	PRJNA1049393	SRR27110007	Genome isolate	Human stool
21EPA039CP01	PRJNA1049393	SRR27110005	Genome isolate	Human stool
21EPA039CP05	PRJNA1049393	SRR27110004	Genome isolate	Human stool
21EPA039CP06	PRJNA1049393	SRR27110003	Genome isolate	Human stool
21EPA039CP08	PRJNA1049393	SRR27110002	Genome isolate	Human stool
21EPA039CP12	PRJNA1049393	SRR27110001	Genome isolate	Human stool
21EPA041CF02	PRJNA1049393	SRR27110000	Genome isolate	Human stool
21EPA041CF03	PRJNA1049393	SRR27109999	Genome isolate	Human stool
21EPA041CP04	PRJNA1049393	SRR27109998	Genome isolate	Human stool
21EPA049CP03	PRJNA1049393	SRR27109997	Genome isolate	Human stool
22EPA077CP09	PRJNA1049393	SRR27109996	Genome isolate	Human stool
22EPA077CP10	PRJNA1049393	SRR27109994	Genome isolate	Human stool
22EPA078CP08	PRJNA1049393	SRR27109993	Genome isolate	Human stool

22EPA079CP08	PRJNA1049393	SRR27109992	Genome isolate	Human stool
22EPA079CP16	PRJNA1049393	SRR27109991	Genome isolate	Human stool
22EPA079CP20	PRJNA1049393	SRR27109990	Genome isolate	Human stool
22EPA080CP5	PRJNA1049393	SRR27109989	Genome isolate	Human stool
22EPA083CP08	PRJNA1049393	SRR27109988	Genome isolate	Human stool
22EPA085CP02	PRJNA1049393	SRR27109987	Genome isolate	Human stool
22EPA085CP05	PRJNA1049393	SRR27109986	Genome isolate	Human stool
22EPA085CP09	PRJNA1049393	SRR27109985	Genome isolate	Human stool
22EPA085CP17	PRJNA1049393	SRR27109983	Genome isolate	Human stool
22EPA087CP04	PRJNA1049393	SRR27109982	Genome isolate	Human stool
22EPA087CP06	PRJNA1049393	SRR27109981	Genome isolate	Human stool
22EPA087CP08	PRJNA1049393	SRR27109980	Genome isolate	Human stool
22EPA088CP02	PRJNA1049393	SRR27109979	Genome isolate	Human stool
22EPA097CP10	PRJNA1049393	SRR27109978	Genome isolate	Human stool
20EPA012CF09	PRJNA1050048	SRR27143334	Genome isolate	Human stool

Table S2. List of gastroenteritis stool samples collected in Norfolk, UK between 2020 and 2022 and the detection of *Campylobacter* by method type

Sample number	Sample ID	Collection year	Bristol scale	Rapid PCR	qPCR results (*cadF* gene detected)	Direct culture
1	20EPA011C	2020	7	Ca+	CA+	CA+
2	20EPA013C	2020	5	Ca+	CA-	CA-
3	20EPA014C	2020	5	Ca+	CA-	CA-
4	20EPA015C	2020	6	Ca+	CA+	CA+
5	20EPA017C	2020	7	Ca+	CA+	CA+
6	20EPA026C	2020	7	Ca+	CA+	CA+
7	20EPA027C	2020	3	Ca+	CA+	CA+
8	20EPA028C	2020	5	Ca+	CA+	CA-
9	21EPA034C	2021	7	Ca+	CA+	CA+
10	21EPA035C	2021	5	Ca+	CA+	CA-
11	21EPA036C	2021	5	Ca+	CA+	CA+
12	21EPA037C	2021	7	Ca+	CA+	CA-
13	21EPA039C	2021	5	Ca+	CA+	CA+
14	21EPA041C	2021	7	Ca+	CA+	CA+
15	21EPA042C	2021	7	Ca+	CA+	CA-
16	21EPA048C	2021	5	Ca+	CA-	CA-
17	21EPA049C	2021	6	Ca+	CA-	CA+
18	21EPA050C	2021	5	Ca+	CA-	CA+
19	21EPA051C	2021	6	Ca+	CA-	CA+
20	21EPA052C	2021	4	Ca+	CA-	CA+
21	21EPA054C	2021	5	Ca+	CA-	CA-
22	21EPA055C	2021	4	Ca+	CA-	CA-
23	22EPA078C	2022	7	Ca+	CA+	CA+
24	22EPA079C	2022	5	Ca+	CA+	CA+
25	22EPA080C	2022	6	Ca+	CA+	CA+
26	22EPA083C	2022	7	Ca+	CA+	CA+
27	22EPA070C	2022	6	Ca+	CA+	CA+
28	22EPA081C	2022	5	Ca+	CA+	CA-
29	22EPA085C	2022	6	Ca+	CA+	CA+

30	22EPA086C	2022	7	Ca+	CA+	CA+
31	22EPA087C	2022	6	Ca+	CA+	CA+
32	22EPA088C	2022	7	Ca+	CA+	CA+
33	22EPA089C	2022	7	Ca+	CA+	CA+
34	22EPA091C	2022	7	Ca+	CA+	CA-
35	22EPA094C	2022	6	Ca+	CA+	CA-
36	22EPA097C	2022	7	Ca+	CA+	CA-
37	22EPA077C	2022	7	Ca+	CA+	CA+
38	20EPA026N	2020	6	Ca-	CA-	CA+
39	21EPA037N	2022	5	Ca-	CA-	CA-
40	22EPA041N	2022	5	Ca-	CA-	CA-
41	22EPA061N	2020	7	Ca-	CA-	CA-
42	22EPA062N	2021	6	Ca-	CA-	CA-

Ca+: *Campylobacter* detected; **Ca-**: *Campylobacter* not detected; **cadF**: *Campylobacter* virulence gene responsible for adhesion used to identify *Campylobacter* genus.

Table S3. Quality of metagenome sequence characteristics from 42 stool samples collected from gastroenteritis cases in Norfolk, UK between August 2020 and June 2022

Rapid PCR detection	Sample type	Sample number	Sample ID	*Campylobacter* CT value	Total reads number	N50 [bp]	Number of *Campylobacter* reads	Percentage of *Campylobacter* reads out of total reads sequenced	*Campylobacter* genome completeness percentage	Coverage [X]
		14	21EPA041CP	31.01	20,327,972	54,300	1,860,000	9.15%	99.6	164.11
		17	21EPA049CF	Not detected	204,533,866	112,000	15261122	7.46%	99.6	1346
		27	22EPA070CP	26.11	24,453,728	56,331	339,468	1.39%	99.7	29.95
		26	22EPA083CP	24.63	141,099,522	184,431	1,683,628	1.19%	99.7	148.55
		35	22EPA094CP	26.53	45,455,312	56121	366,350	0.81%	98.99	38.27
		7	20EPA027CP	21	53,550,853	14312	428,406	0.80%	99.3	37.8
		22	21EPA055CF	Not detected	25,546,072	700	170,344	0.67%	91.8	15.03
		23	22EPA078CP	24.88	149,948,610	57,688	921,158	0.61%	99.7	81.27
		19	21EPA051CF	35.06	22,289,818	800	123,352	0.55%	42	10.88
		4	20EPA015CP	18.09	71,043,814	1608	355,219	0.50%	0.922	31.34
		25	22EPA080CP	24.79	192,151,666	66,862	864,736	0.45%	99.7	76.3
		16	21EPA048CF	Not detected	31,846,892	500	110,364	0.35%	-	9.73
		24	22EPA079CP	26.8	44,831,110	3,418	135,104	0.30%	62.7	11.92
		21	21EPA054CF	Not detected	23,067,429	600	61,692	0.27%	7.3	5.44
		18	21EPA050CF	Not detected	65,571,000	1900	164,758	0.25%	87.5	14.53
		13	21EPA039CF	29	103,188,204	15445	258,498	0.25%	92.45	22.8
		31	22EPA087CP	24.08	97,171,704	196485	235,457	0.24%	99.44	33.67
		22	21EPA055CP	26.3	30,162,254	3300	68,396	0.23%	21.9	6.03
		5	20EPA017CP	19	88,670,850	3884	182,554	0.21%	91.52	16.1
	Filtered (n=10)	1	20EPA011CF	19.34	145,701,074	2810	291,402	0.20%	96.1	25.7
		3	20EPA014CP	Not detected	51,313,672	639	102,627	0.20%	-	9.05
		18	21EPA050CP	38.2	25,380,113	-	48,612	0.19%	5	4.28
		9	21EPA034CF	28	210,406,752	89548	377,406	0.18%	92.1	33.3
		20	21EPA052CF	40	53,560,711	1,000	61156	0.11%	47.2	5.39
		36	22EPA097CP	25.84	1,971,022	8049	2,151	0.11%	92.92	39.27
		11	21EPA036CP	24	88,315,068	1247	56,628	0.06%	36.38	4.99

		16	21EPA048CP	35.94	49,972,087	400	25,976	0.05%	-	2.29
		19	21EPA051CP	30.58	23,692,918	800	12,060	0.05%	-	1.06
		21	21EPA054CP	28.42	30,127,255	400	11,836	0.04%	-	1.04
		29	22EPA085CP	21.5	85,238,166	81,281	30,512	0.04%	93.54	33.47
		9	21EPA034CP	23	96,781,602	676	19,688	0.02%	-	1.73
PCR+ (n=37)	Unfiltered (n=35)	2	20EPA013CP	Not detected	30,303,347	-	6,060	0.02%	-	0.53
		37	22EPA077CP	23.83	72,235,446	17221	12,862	0.02%	99.15	49.16
		28	22EPA081CP	29.27	77,134,074	-	11,867	0.02%	-	-
		13	21EPA039CP	34.97	154,327,436	479	15,670	0.01%	0.31	1.38
		8	20EPA028CP	27	86,812,316	399	8,681	0.01%	2.5	76
		15	21EPA042CP	34.4	27,626,330	400	2,422	0.01%	-	0.21
		32	22EPA088CP	25.29	51,073,294	12744	4,428	0.01%	95.79	39.97
		30	22EPA086CP	28.59	43,128,470	-	2,706	0.01%	-	-
	Filtered (n=1)	39	21EPA037NF	Not detected	176,310,814	-	8,903	0.01%	-	-
		6	20EPA026CP	28.95	177,427,648	2598	7,694	0.00%	-	0.67
		40	22EPA041NP	Not detected	30,480,126	-	1,313	0.00%	-	-
		34	22EPA091CP	27.29	68,530,340	1349	2,005	0.00%	7.96	3.26
		42	22EPA062NP	Not detected	65,263,260	-	1,547	0.00%	-	-
		10	21EPA035CP	34	79,674,220	527	1,650	0.00%	-	0.14
PCR- (n=5)	Unfiltered (n=4)	38	20EPA026NP	33	228,671,258	-	2,076	0.00%	-	-
		12	21EPA037CP	31	110,375,488	667	962	0.00%	0	0.08
		33	22EPA089CP	30.35	85,171,086	1349	460	0.00%	-	-
		17	21EPA049CP	28.41	171,254,784	-	734	0.00%	-	-
		41	22EPA061NP	Not detected	67,653,232	-	94	0.00%	-	-

CT: qPCR cycle threshold ; **N50**: assembly contiguity; **bp**: base pairs

Table S4. Description of key *Campylobacter* characterisation from metagenome derived genomes of unfiltered and filtered stool samples that passed quality check for direct sequencing with corresponding same sample isolate derived genomes

Sample ID	Bristol scale	Genome origin	N50	Number of *Campylobacter* reads	Percentage of *Campylobacter* reads in total sequencing	Percentage of genome completeness	Coverage [X]	Species	ST	AMR determinants profile
20EPA011CF	7	Metagenome	2,810	291,402	0.2	96.1	25.7	*C. jejuni*	61	-
20EPA011CF03	7	Genome	-	-	-	-	-	*C. jejuni*	61	blaOXA-193
20EPA011CF04	7	Genome	-	-	-	-	-	*C. jejuni*	61	blaOXA-193
20EPA011CF07	7	Genome	-	-	-	-	-	*C. jejuni*	61	blaOXA-193
20EPA011CF14	7	Genome	-	-	-	-	-	*C. jejuni*	61	blaOXA-193
20EPA011CF15	7	Genome	-	-	-	-	-	*C. jejuni*	61	blaOXA-193
20EPA017CF02	7	Genome	-	-	-	-	-	*C. jejuni*	5136	50S_L22_A103V, blaOXA-193, arc3, gyrA_T86I, tet(O)
20EPA017CF03	7	Genome	-	-	-	-	-	*C. jejuni*	5136	50S_L22_A103V, blaOXA-193, arc3, gyrA_T86I, tet(O)
20EPA017CF04	7	Genome	-	-	-	-	-	*C. jejuni*	5136	50S_L22_A103V, blaOXA-193, arc3, gyrA_T86I, tet(O)
20EPA017CF06	7	Genome	-	-	-	-	-	*C. jejuni*	5136	50S_L22_A103V, blaOXA-193, arc3, gyrA_T86I, tet(O)
20EPA017CF07	7	Genome	-	-	-	-	-	*C. jejuni*	5136	50S_L22_A103V, blaOXA-193, arc3, gyrA_T86I, tet(O)
20EPA017CF08	7	Genome	-	-	-	-	-	*C. jejuni*	5136	50S_L22_A103V, blaOXA-193, arc3, gyrA_T86I, tet(O)
20EPA017CF09	7	Genome	-	-	-	-	-	*C. jejuni*	5136	50S_L22_A103V, blaOXA-193, arc3, gyrA_T86I, tet(O)
20EPA017CF10	7	Genome	-	-	-	-	-	*C. jejuni*	5136	50S_L22_A103V, blaOXA-193, arc3, gyrA_T86I, tet(O)
20EPA017CF11	7	Genome	-	-	-	-	-	*C. jejuni*	5136	50S_L22_A103V, blaOXA-193, arc3, gyrA_T86I, tet(O)
20EPA017CF14	7	Genome	-	-	-	-	-	*C. jejuni*	5136	50S_L22_A103V, blaOXA-193, arc3, gyrA_T86I, tet(O)
20EPA017CP	7	Metagenome	3884	182,554	0.206	91.52	16.1	*C. jejuni*	5136	50S_L22_A103V, arc3, gyrA_T86I
20EPA017CP01	7	Genome	-	-	-	-	-	*C. jejuni*	5136	50S_L22_A103V, blaOXA-193, arc3, gyrA_T86I, tet(O)
20EPA017CP03	7	Genome	-	-	-	-	-	*C. jejuni*	5136	50S_L22_A103V, blaOXA-193, arc3, gyrA_T86I, tet(O)
20EPA017CP06	7	Genome	-	-	-	-	-	*C. jejuni*	5136	50S_L22_A103V, blaOXA-193, arc3, gyrA_T86I, tet(O)
20EPA017CP07	7	Genome	-	-	-	-	-	*C. jejuni*	5136	50S_L22_A103V, blaOXA-193, arc3, gyrA_T86I, tet(O)
20EPA017CP09	7	Genome	-	-	-	-	-	*C. jejuni*	5136	50S_L22_A103V, blaOXA-193, arc3, gyrA_T86I, tet(O)
20EPA017CP10	7	Genome	-	-	-	-	-	*C. jejuni*	5136	50S_L22_A103V, blaOXA-193, arc3, gyrA_T86I, tet(O)
20EPA017CP11	7	Genome	-	-	-	-	-	*C. jejuni*	5136	50S_L22_A103V, blaOXA-193, arc3, gyrA_T86I, tet(O)
20EPA017CP12	7	Genome	-	-	-	-	-	*C. jejuni*	5136	50S_L22_A103V, blaOXA-193, arc3, gyrA_T86I, tet(O)
20EPA017CP14	7	Genome	-	-	-	-	-	*C. jejuni*	5136	50S_L22_A103V, blaOXA-193, arc3, gyrA_T86I, tet(O)

20EPA027CF03	3	Genome	-	-	-	-	-	*C. coli*	827	50S_L22_A103V, arc3, blaOXA-489
20EPA027CF08	3	Genome	-	-	-	-	-	*C. coli*	827	50S_L22_A103V, arc3, blaOXA-489
20EPA027CP	3	Metagenome	14312	428,406	0.8	99.3	37.8	*C. coli*	827	50S_L22_A103V, arc3, blaOXA-489
21EPA034CF	7	Metagenome	676	19,688	0.02	0	1.73	*C. jejuni*	9897	blaOXA-193, gyrA_T86I
21EPA034CF07	7	Genome	-	-	-	-	-	*C. jejuni*	9897	blaOXA-193, gyrA_T86I, tet(O)
21EPA034CF14	7	Genome	-	-	-	-	-	*C. jejuni*	9897	blaOXA-193, gyrA_T86I, tet(O)
21EPA039CF	5	Metagenome	15445	258,498	0.251	92.45	22.8	*C. jejuni*	45	acr3, blaOXA
21EPA039CF01	5	Genome	-	-	-	-	-	*C. jejuni*	45	acr3, blaOXA-193
21EPA039CF08	5	Genome	-	-	-	-	-	*C. jejuni*	45	acr3, blaOXA-193
21EPA039CF11	5	Genome	-	-	-	-	-	*C. jejuni*	45	acr3, blaOXA-193
21EPA039CF13	5	Genome	-	-	-	-	-	*C. jejuni*	45	acr3, blaOXA-193
21EPA039CF14	5	Genome	-	-	-	-	-	*C. jejuni*	45	acr3, blaOXA
21EPA039CF15	5	Genome	-	-	-	-	-	*C. jejuni*	45	acr3, blaOXA
21EPA039CP01	5	Genome	-	-	-	-	-	*C. jejuni*	45	acr3, blaOXA
21EPA039CP05	5	Genome	-	-	-	-	-	*C. jejuni*	45	acr3, blaOXA-193
21EPA039CP06	5	Genome	-	-	-	-	-	*C. jejuni*	45	acr3, blaOXA
21EPA039CP08	5	Genome	-	-	-	-	-	*C. jejuni*	45	acr3, blaOXA-193
21EPA039CP12	5	Genome	-	-	-	-	-	*C. jejuni*	45	acr3, blaOXA
21EPA041CF02	7	Genome	-	-	-	-	-	*C. jejuni*	9897	blaOXA-193, gyrA_T86I
21EPA041CF03	7	Genome	-	-	-	-	-	*C. jejuni*	9897	blaOXA-193, gyrA_T86I
21EPA041CP	7	Metagenome	54,300	1,860,000	9.15	99.6	164.11	*C. jejuni*	9897	blaOXA-193, gyrA_T86I
21EPA041CP04	7	Genome	-	-	-	-	-	*C. jejuni*	-	blaOXA-193, gyrA_T86I
21EPA049CF	6	Metagenome	112,000	15261122	7.461	99.6	1346	*C. jejuni*	38	blaOXA-193
21EPA049CP03	6	Genome	-	-	-	-	-	*C. jejuni*	38	blaOXA-193
22EPA077CP	6	Metagenome	17221	12,862	0.018	99.15	49.16	*C. jejuni*	464	50S_L22_A103V, arcr3, arsP, blaOXA-193, gyrA_T86I
22EPA077CP09	6	Genome	-	-	-	-	-	*C. jejuni*	464	_L22_A103V, arcr3, arsP, blaOXA-193, gyrA_T86I, tet(O)
22EPA077CP10	6	Genome	-	-	-	-	-	*C. jejuni*	464	_L22_A103V, arcr3, arsP, blaOXA-193, gyrA_T86I, tet(O)
22EPA078CP	7	Metagenome	57,688	921,158	0.614	99.7	81.27	*C. jejuni*	137	blaOXA-193
22EPA078CP08	7	Genome	-	-	-	-	-	*C. jejuni*	137	blaOXA-193
22EPA079CP	5	Metagenome	3,418	135,104	0.301	62.7	11.92	*C. jejuni*	-	blaOXA
22EPA079CP08	5	Genome	-	-	-	-	-	*C. jejuni*	50	arsP, blaOXA-193
22EPA079CP16	5	Genome	-	-	-	-	-	*C. jejuni*	50	arsP, blaOXA-193
22EPA079CP20	5	Genome	-	-	-	-	-	*C. jejuni*	50	arsP, blaOXA-193

22EPA080CP	6	Metagenome	66,862	864,736	0.45	99.7	76.3	*C. jejuni*	441	blaOXA-193, gyrA_P104S, gyrA_T86I
22EPA080CP5	6	Genome	-	-	-	-	-	*C. jejuni*	441	blaOXA-193, gyrA_P104S, gyrA_T86I
22EPA083CP	7	Metagenome	184,431	1,683,628	1.193	99.7	148.55	*C. jejuni*	6532	blaOXA-193, gyrA_T86I
22EPA083CP08	7	Genome	-	-	-	-	-	*C. jejuni*	6532	aadE, blaOXA-193, gyrA_T86I
22EPA085CP	7	Metagenome	81,281	30,512	0.036	93.54	33.47	*C. jejuni*	-	arc3, blaOXA-61
22EPA085CP02	7	Genome	-	-	-	-	-	*C. jejuni*	21	blaOXA-193
22EPA085CP09	7	Genome	-	-	-	-	-	*C. jejuni*	48	arc3, blaOXA-61
22EPA085CP17	7	Genome	-	-	-	-	-	*C. jejuni*	48	arc3, blaOXA-61
22EPA087CP	7	Metagenome	196485	235,457	0.242	99.44	33.67	*C. coli*	1055	arc3, tet(O)
22EPA087CP04	7	Genome	-	-	-	-	-	*C. coli*	1055	arc3, tet(O)
22EPA087CP06	7	Genome	-	-	-	-	-	*C. coli*	1055	arc3, tet(O)
22EPA087CP08	7	Genome	-	-	-	-	-	*C. coli*	1055	arc3, tet(O)
22EPA088CP	7	Metagenome	12744	4,428	0.009	95.79	39.97	*C. jejuni*	883	arsP, blaOXA, gyrA_T86I
22EPA088CP02	7	Genome	-	-					447	arsP
22EPA097CP	7	Metagenome	8049	2,151	0.109	92.92	39.27	*C. jejuni*	6175	blaOXA-193, gyrA_T86I, tet(O)
22EPA097CP10	7	Genome	-	-	-	-	-	*C. jejuni*	6175	blaOXA-193, gyrA_T86I, tet(O)

ST: sequence type ; **AMR**: antimicrobial resistance gene determinant ; **-**: not detected ; bla: beta lactamase ; **tet**: tetracycline ; **gyr**: gyrase ; **P in sample ID**: Unfiltered sample type ; **F in sample ID**: filtered sample type.

Table S5. BUSCO and MLST report on *Campylobacter* genomes assebled from stool metagenomes spiked with varied *Campylobacter* genome levels: a comparison of standard and adaptive sequencing settings on MinION ONT sequencer platform

**Sample + *Campylobacter* %**	**Sequencer setting**	**Complete BUSCOs (C) percentage**	**Complete and single-copy BUSCOs (S) percentage**	**Complete and duplicated BUSCOs (D) percentage**	**Fragmented BUSCOs (F) percentage**	**Missing BUSCOs (M) percentage**	**Total BUSCO groups searched**	**ST**	**Seven MLST housekeeping genes**						
									**glnA**	**glnA**	**gltA**	**glyA**	**pgm**	**tkt**	**uncA**
Stool stock + 2%	A	93.5	93.5	0	0.5	6.0	628	2066	9	10	5	10	22	3	6
	S	92.8	92.8	0	0.6	6.6	628	-	9	10	5	10	22	3	~6
Stool stock + 1%	A	92.4	92.4	0.0	0.4	6.8	628	2066	9	10	5	10	22	3	6
	S	92.2	92.2	0	0.8	7.0	628	2066	9	10	5	10	22	3	6
Stool stock + 0.5%	A	51	40	0	7	77	124	-	-	-	-	10	-	-	-
	S	47	45	10	16	53	124	-	-	-	-	-	-	3	-
Stool stock + 0.1%	A	1.5	-	-	-	-	-	-	-	-	-	-	-	-	-
	S	2.95	-	-	-	-	-	-	-	-	-	-	-	-	-
Stool stock only	A	-	-	-	-	-	-	-	-	-	-	-	-	-	-
	S	-	-	-	-	-	-	-	-	-	-	-	-	-	-
Mock community only	A	-	-	-	-	-	-	-	-	-	-	-	-	-	-
	S	-	-	-	-	-	-	-	-	-	-	-	-	-	-
Mock community + 2%	A	92.7	92.7	0	0.3	7	628	2066	9	10	5	10	22	3	6
	S	93.6	93.6	0	0.3	6.1	628	2066	9	10	5	10	22	3	6
Clinical sample metagenome (22EPA077CP)	A	93.5	93.5	0	4.8	1.7	628	464	24	2	2	2	10	3	1
	S	93.2	93.2	0	3.7	3.1	628	-	24	2	2	2	10	3	~1

ST: sequence type **; A:** Adaptive **; S:** Standard **; MLST:** Multi-locus seqeunce typing
